# Supplementary material for: Biocompatible Ir(III) Complexes as Oxygen Sensors for Phosphorescence Lifetime Imaging
Source: Molecules. 2021 May 13;26(10):2898. doi: 10.3390/molecules26102898 (PMC8153025; doi:10.3390/molecules26102898)
Supplement: Supplementary file 1 [file molecules-26-02898-s001.zip › molecules-1215256-supplementary.pdf]

molecules

Supplementary Material for the article

Biocompatible Ir(III) Complexes as Oxygen Sensors for Phosphorescence Lifetime Imaging

Ilya S. Kritchenkov [1], Anastasia I. Solomatina [1], Daria O. Kozina [1], Vitaly V. Porsev [1], Victor V. Sokolov [1], Marina V. Shirmanova [2], Maria M. Lukina [2], Anastasia D. Komarova [2], Vladislav I. Shcheslavskiy [2,3], Tatiana N. Belyaeva [4], Ilia K. Litvinov [4], Anna V. Salova [4], Elena S. Kornilova [4,5], Daniel V. Kachkin [6] and Sergey P. Tunik [1,*]

[1] Institute of Chemistry, St. Petersburg State University, Universitetskii av., 26, 198504 St. Petersburg, Russia; i.s.kritchenkov@spbu.ru (I.S.K.); nastisol@gmail.com (A.I.S.); st055671@student.spbu.ru (D.O.K.); v.porsev@spbu.ru (V.V.P.); v.sokolov@spbu.ru (V.V.S.)

[2] Institute of Experimental Oncology and Biomedical Technologies, Privolzhskiy Research Medical University, Minin and Pozharsky sq. 10/1, 603005 Nizhny Novgorod, Russia; shirmanovam@gmail.com (M.V.S.); kuznetsova.m.m@yandex.ru (M.M.L.); komarova.anastasii@gmail.com (A.D.K.); vis@becker-hickl.de (V.I.S.)

[3] Becker&Hickl GmbH, Nunsdorfer Ring 7-9, 12277 Berlin, Germany

[4] Institute of Cytology of the Russian Academy of Sciences, Tikhoretsky av. 4, 194064 St. Petersburg, Russia; tatbelyaeva@gmail.com (T.N.B.); lik314@mail.ru (I.K.L.); avsalova@gmail.com (A.V.S.); lenkor@incras.ru (E.S.K.)

[5] Institute of Biomedical Systems and Biotechnology, Peter the Great St. Petersburg Polytechnical University, Khlopina str. 11, 194021 St. Petersburg, Russia

[6] Faculty of Biology, St. Petersburg State University, Universitetskaya emb., 7/9, 199034 St. Petersburg, Russia; pspdaniel@mail.ru

* Correspondence: sergey.tunik@spbu.ru; Tel.: +7 921 3111830

NMR spectroscopy and ESI mass-spectrometry data

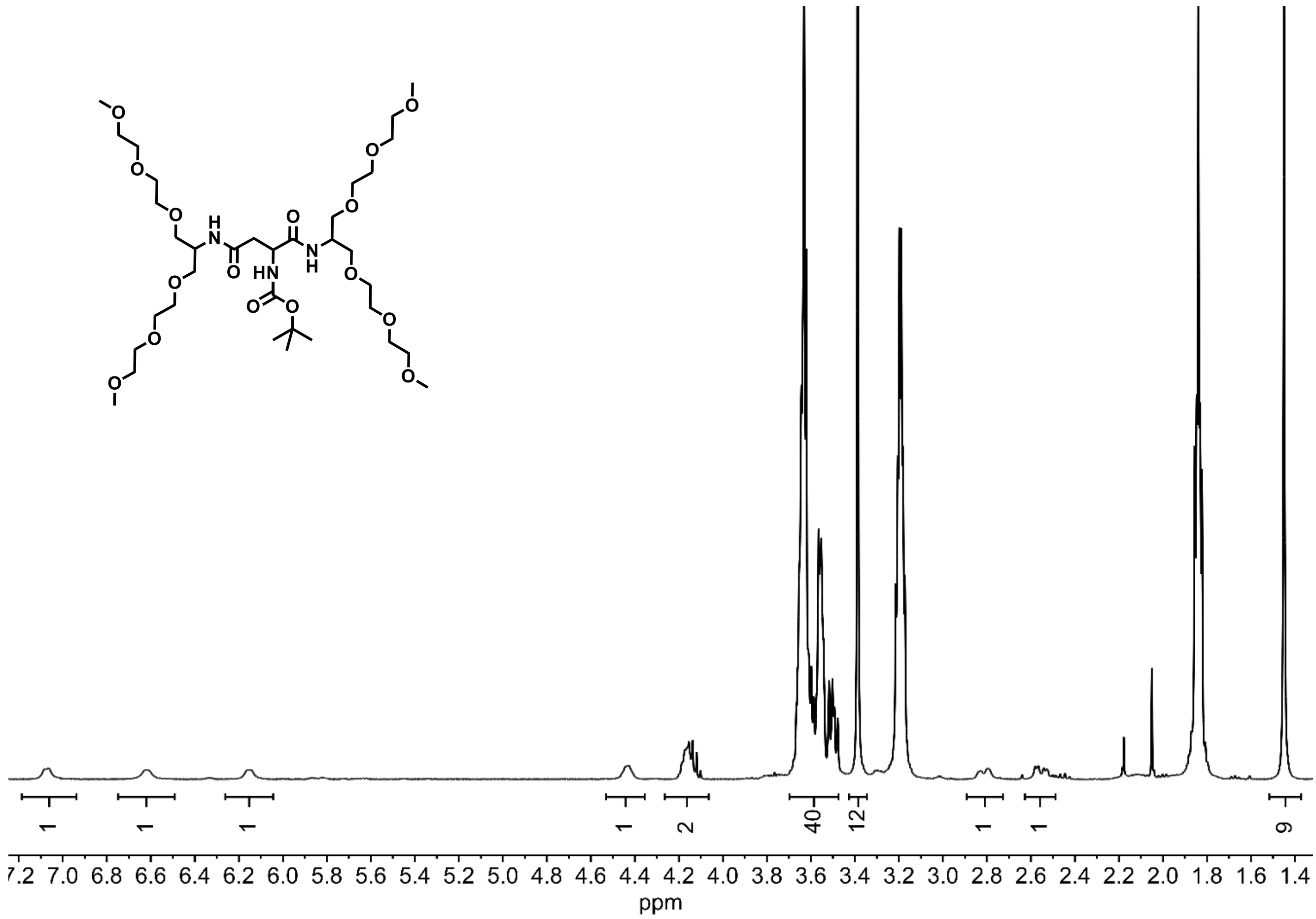

Figure S1. ^{1}H NMR spectrum of **NHBoc-4OEG**, $CDCl_3$, 298 K.

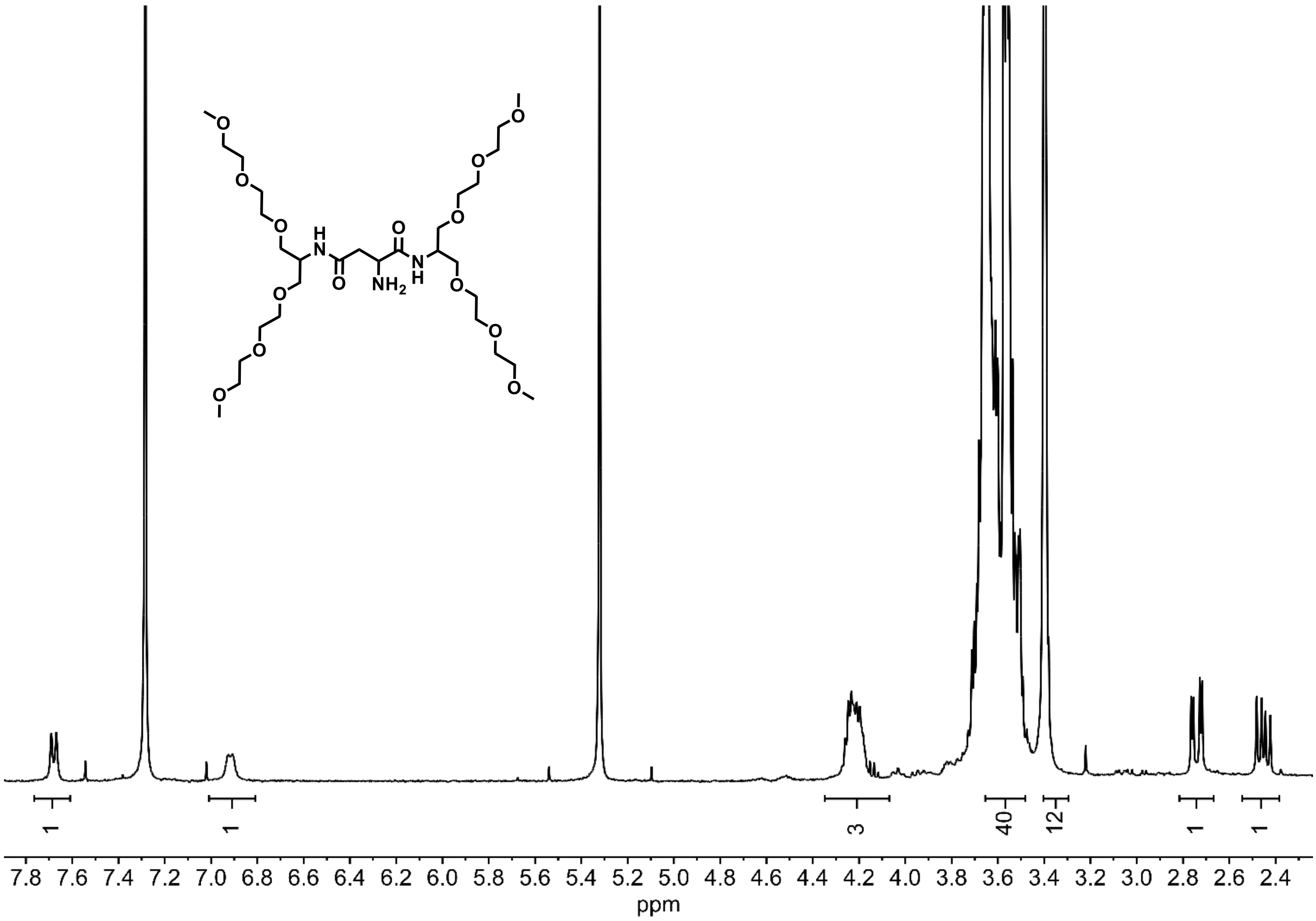

Figure S2. ^{1}H NMR spectrum of **NH_2-4OEG**, $CDCl_3$, 298 K.

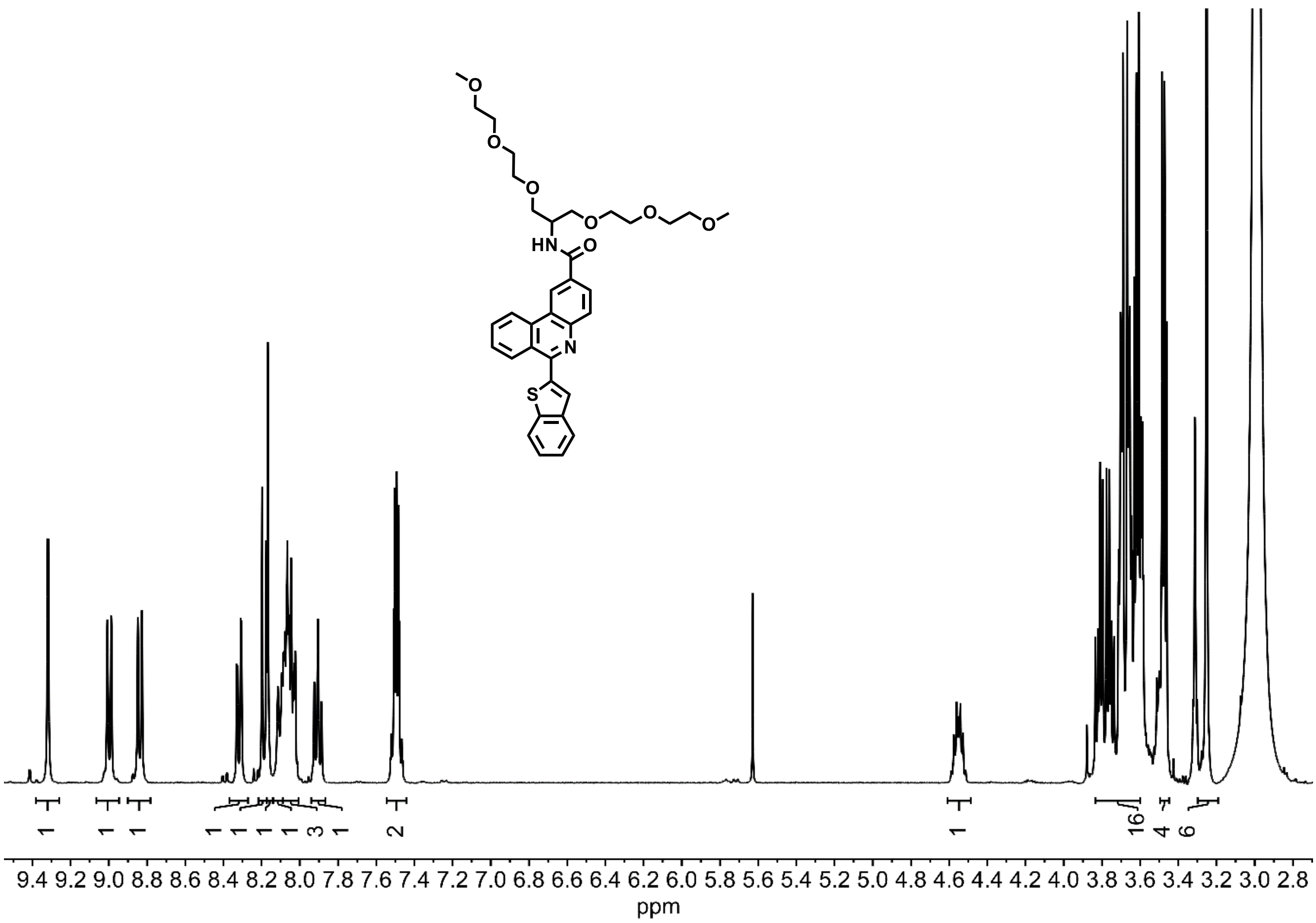

Figure S3. ^{1}H NMR spectrum of **N^C1**, $(CD_3)_2CO$, 298 K.

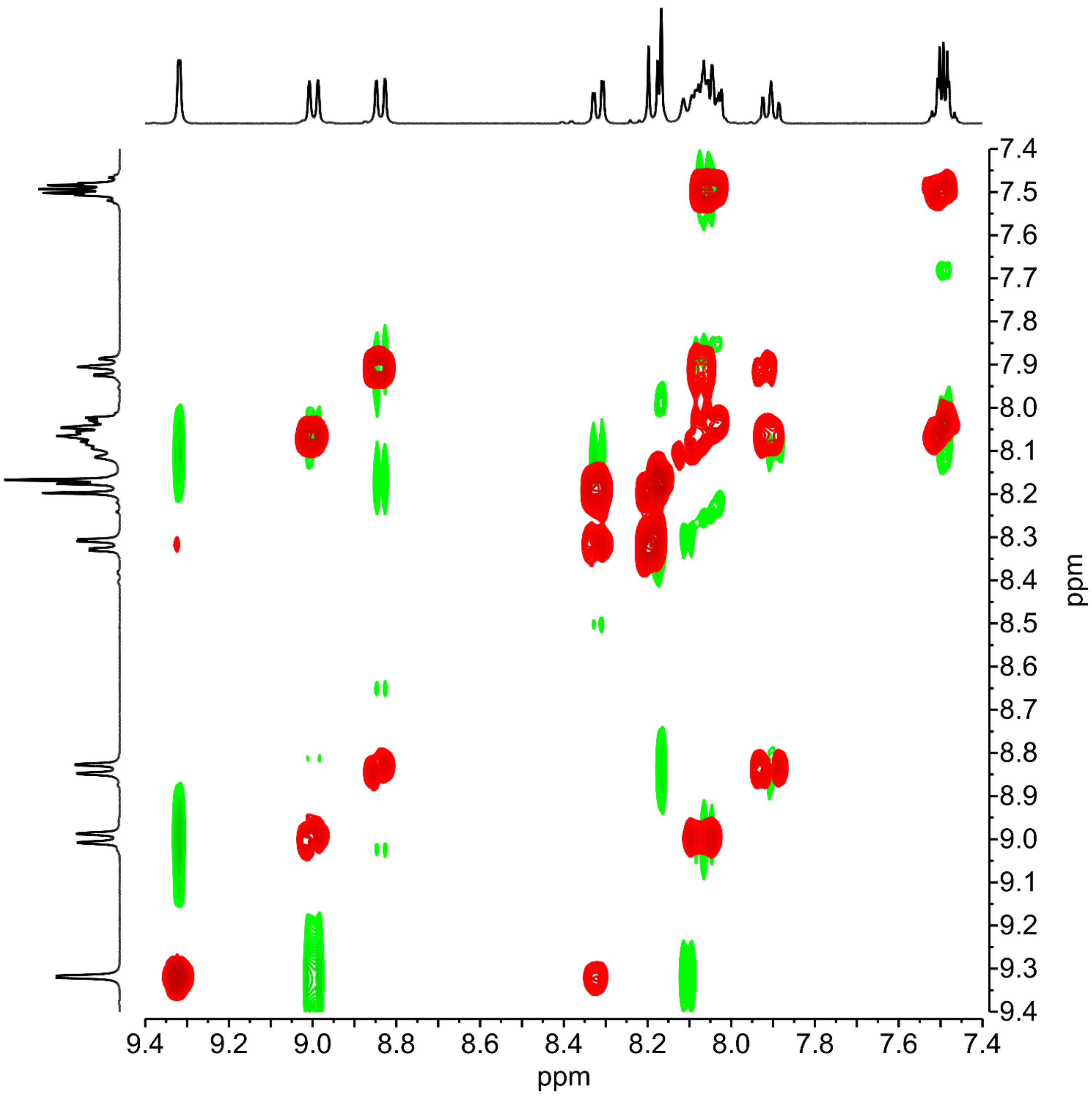

Figure S4. ^{1}H-^{1}H COSY and NOESY NMR spectra of **N^C1**, $(CD_3)_2CO$, 298 K. Red diagonal and crosspeaks are from COSY spectrum, green crosspeaks are from NOESY spectrum.

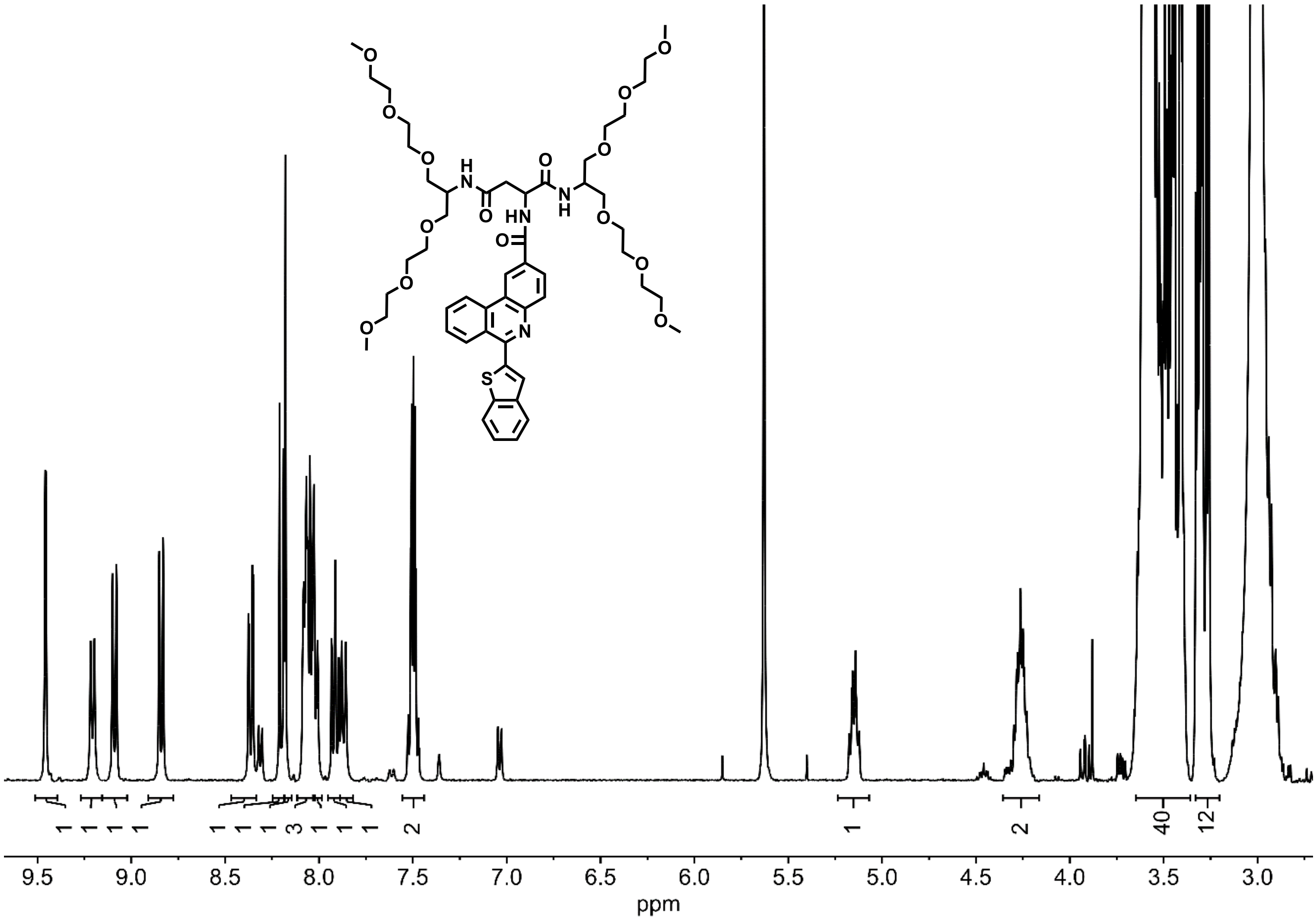

Figure S5. ^{1}H NMR spectrum of **N^C2**, $(CD_3)_2CO$, 298 K.

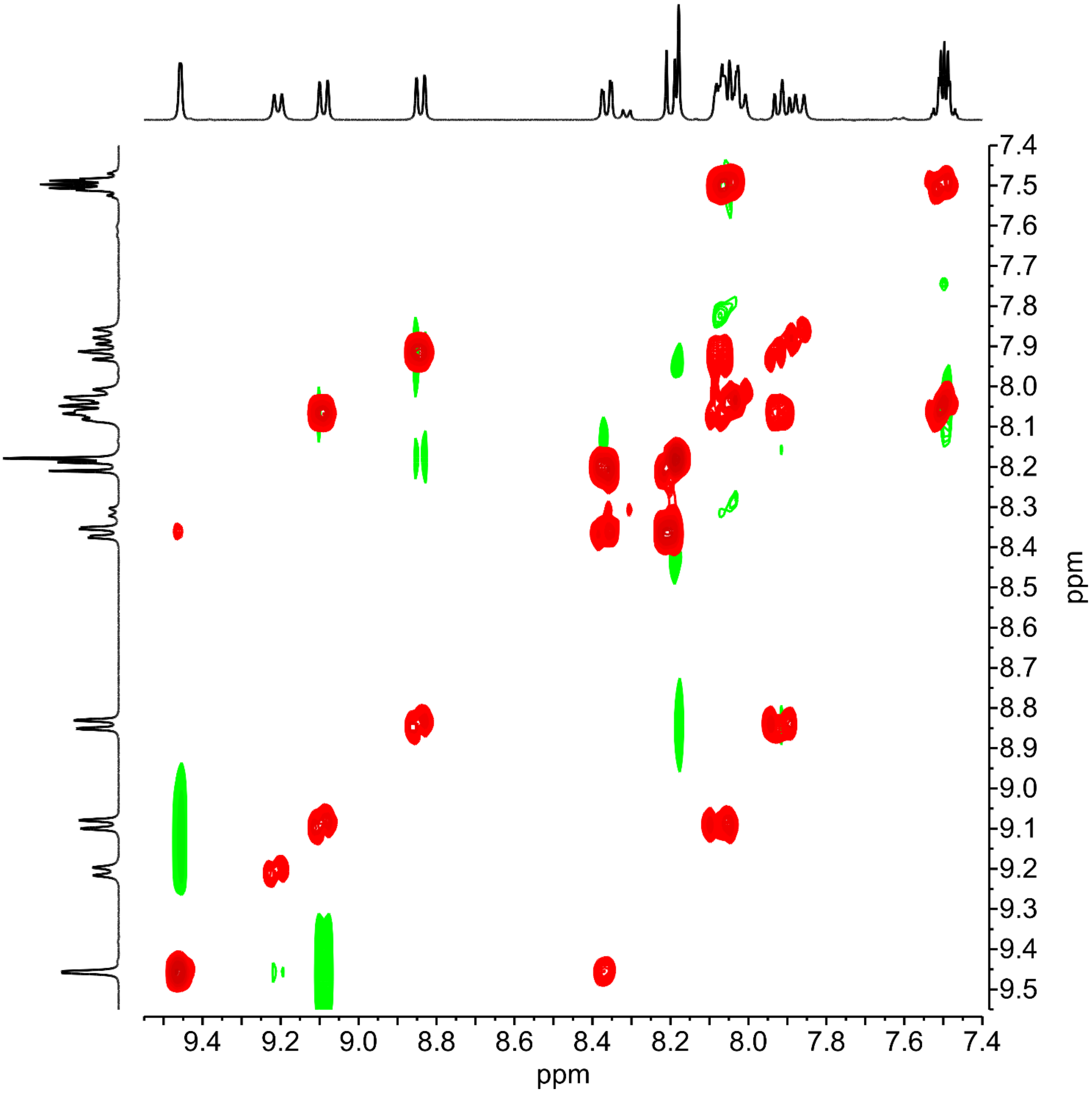

Figure S6. ^{1}H-^{1}H COSY and NOESY NMR spectra of **N^C2**, $(CD_3)_2CO$, 298 K. Red diagonal and crosspeaks are from COSY spectrum, green crosspeaks are from NOESY spectrum.

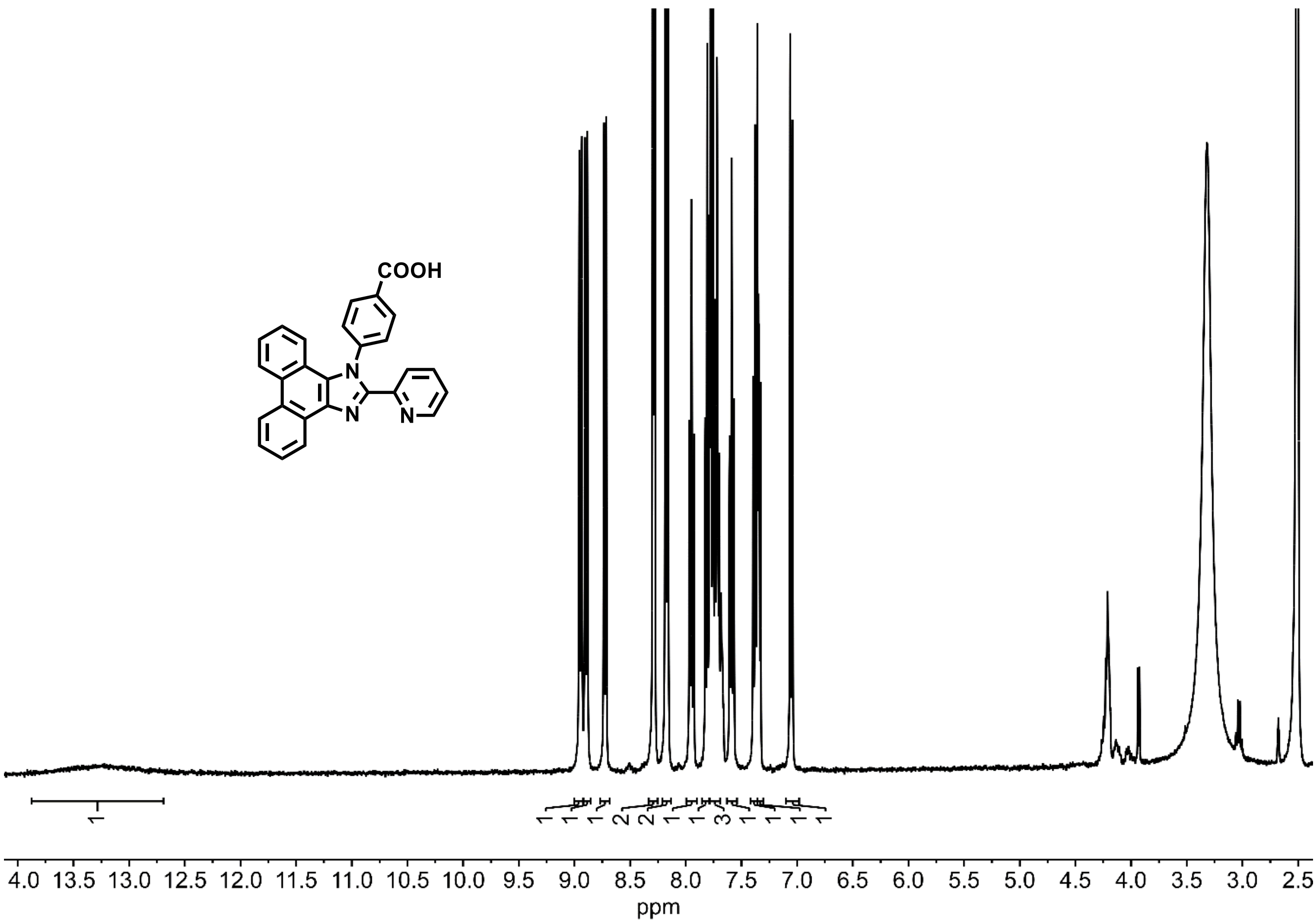

Figure SS7. ^{1}H NMR spectrum of 4-(2-(pyridin-2-yl)-1H-phenanthro[9,10-d]imidazol-1-yl)benzoic acid, $(CD_3)_2SO$, 298 K.

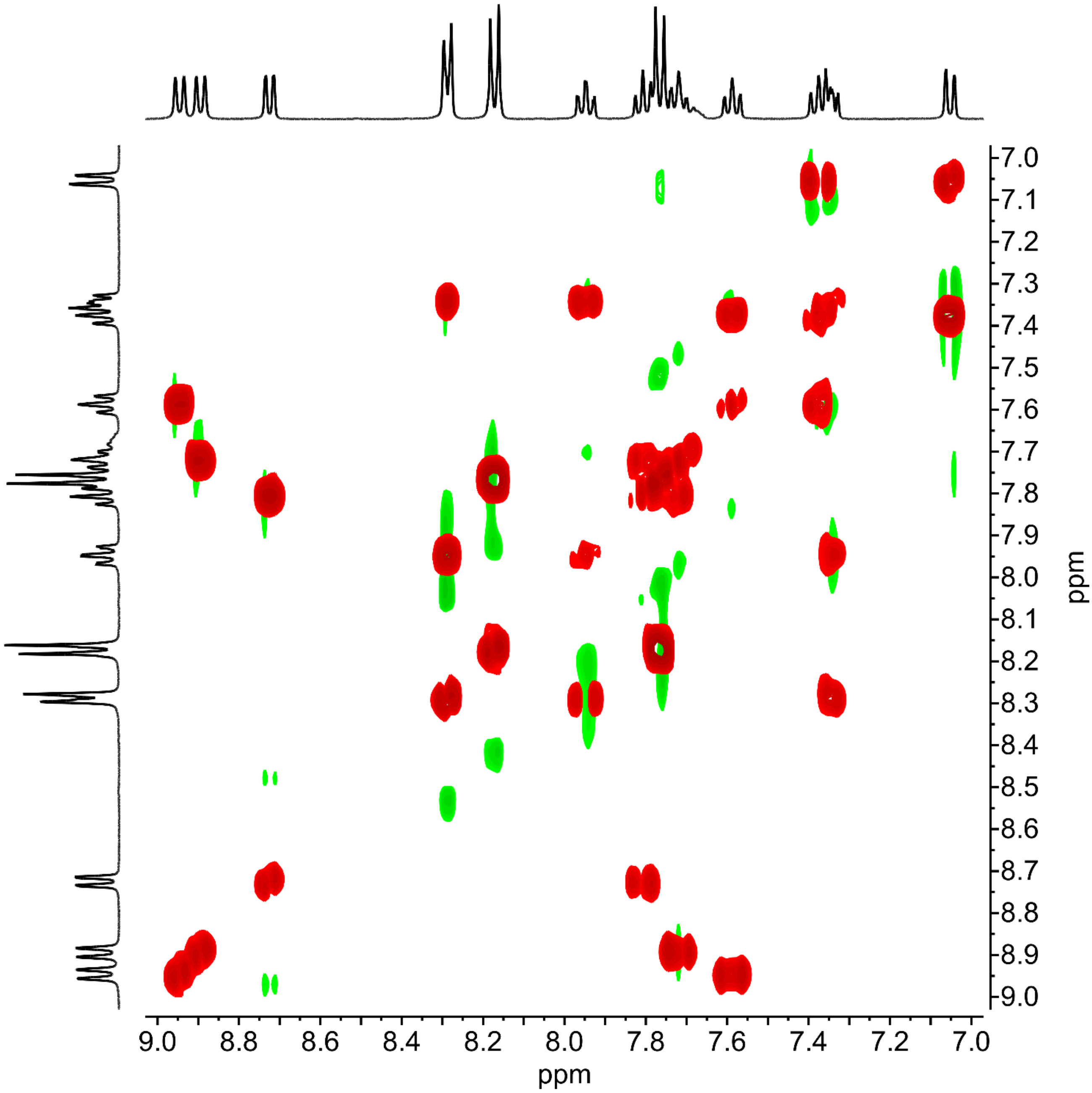

Figure S8. ^{1}H-^{1}H COSY and NOESY NMR spectra of **4-(2-(pyridin-2-yl)-1H-phenanthro[9,10-d]imidazol-1-yl)benzoic acid**, $(CD_3)_2SO$, 298 K. Red diagonal and crosspeaks are from COSY spectrum, green crosspeaks are from NOESY spectrum.

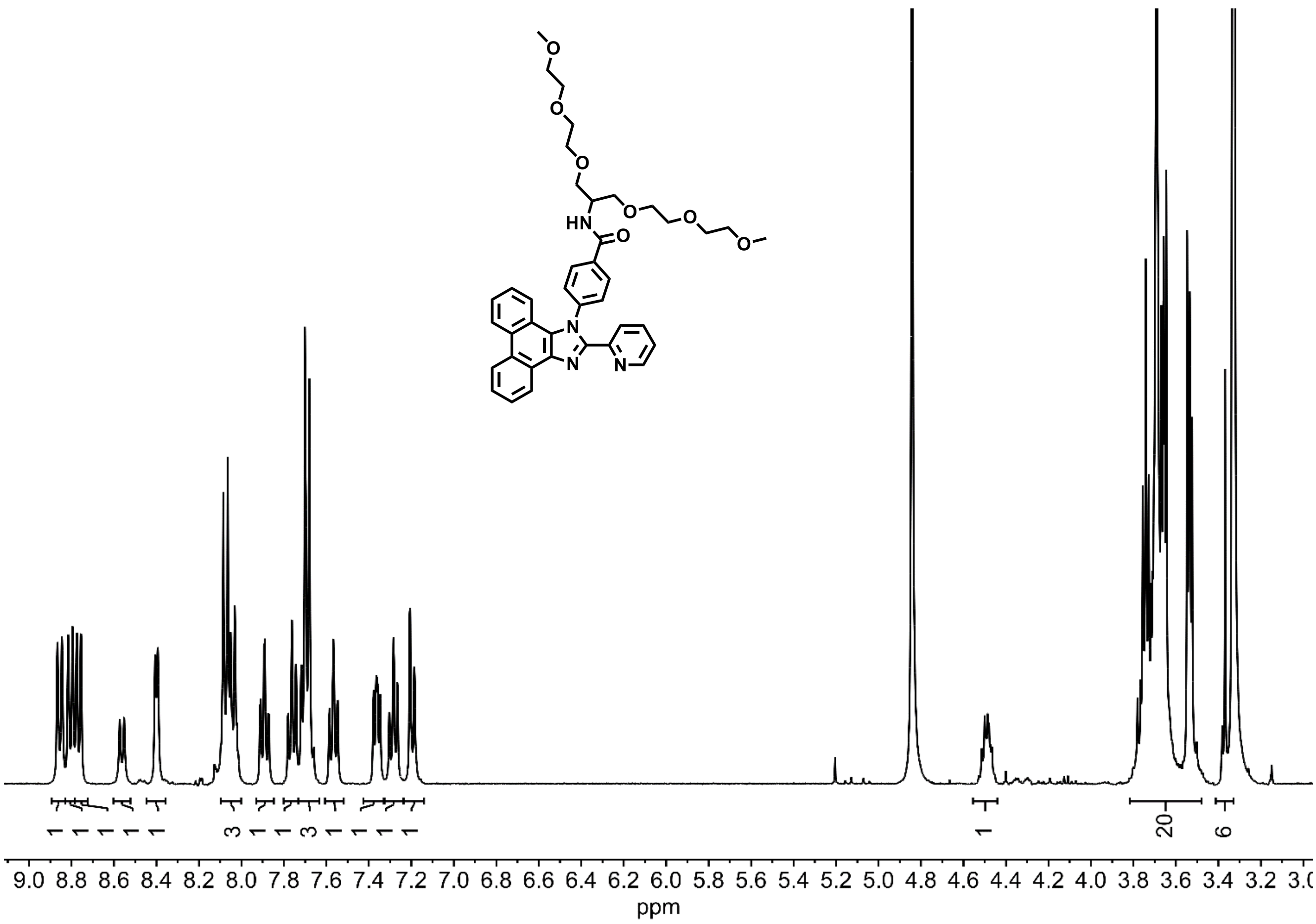

Figure S9. ^{1}H NMR spectrum of **N^N1**, CD_3OD, 298 K.

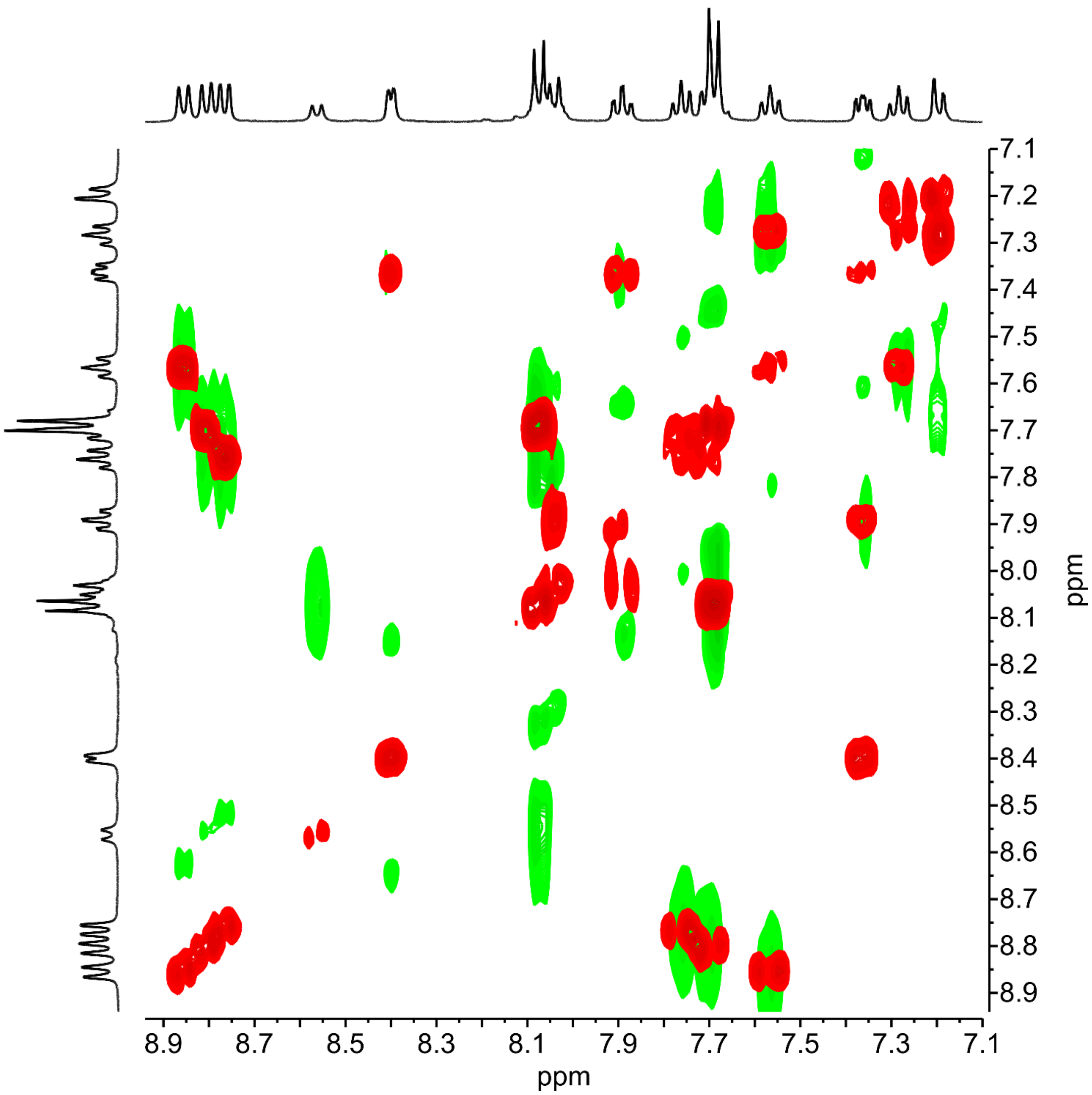

Figure S10. ^1H-^1H COSY and NOESY NMR spectra of **N^N1**, CD_3OD, 298 K. Red diagonal and crosspeaks are from COSY spectrum, green crosspeaks are from NOESY spectrum.

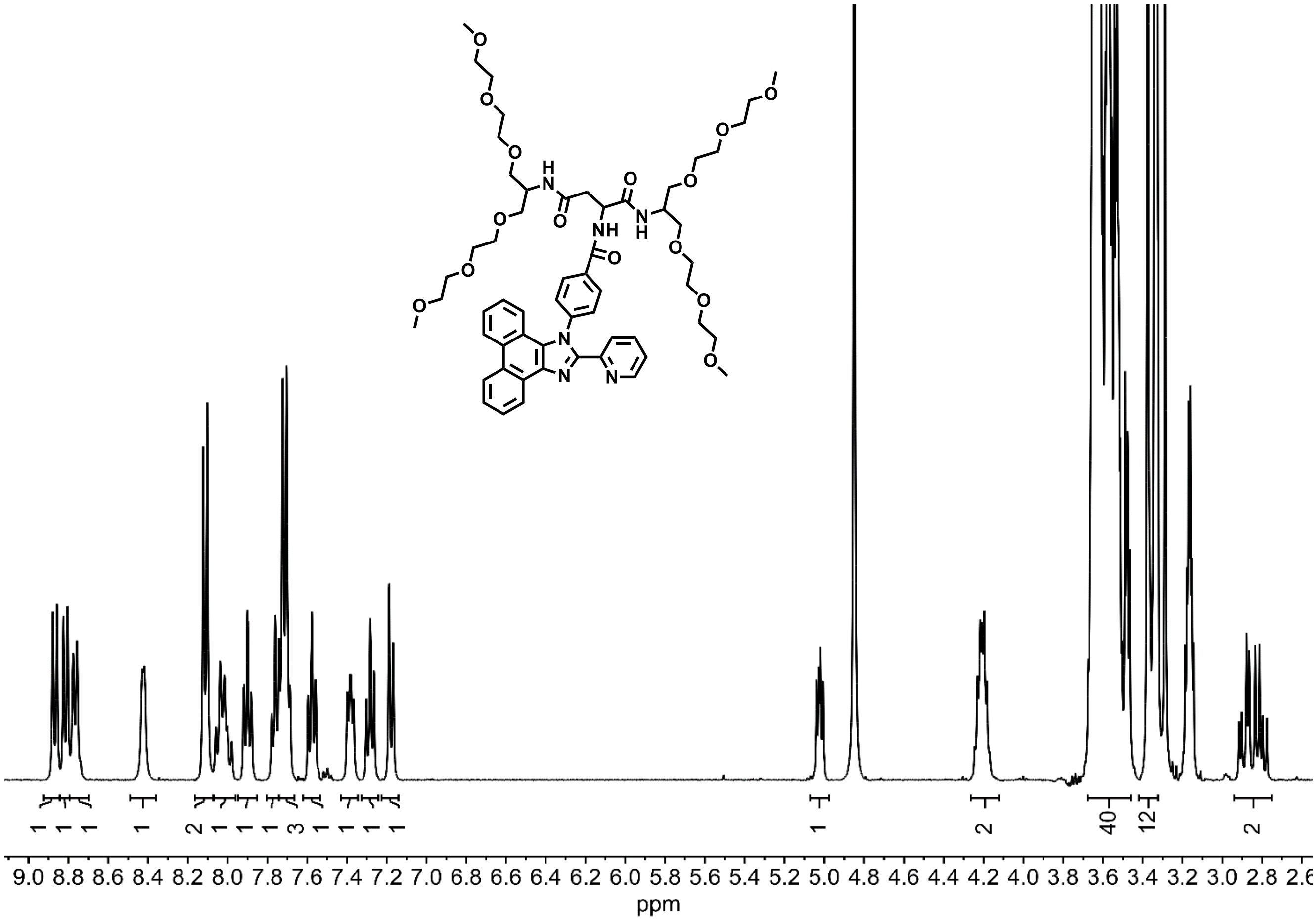

Figure S11. ^{1}H NMR spectrum of **N^N2**, CD_3OD, 298 K.

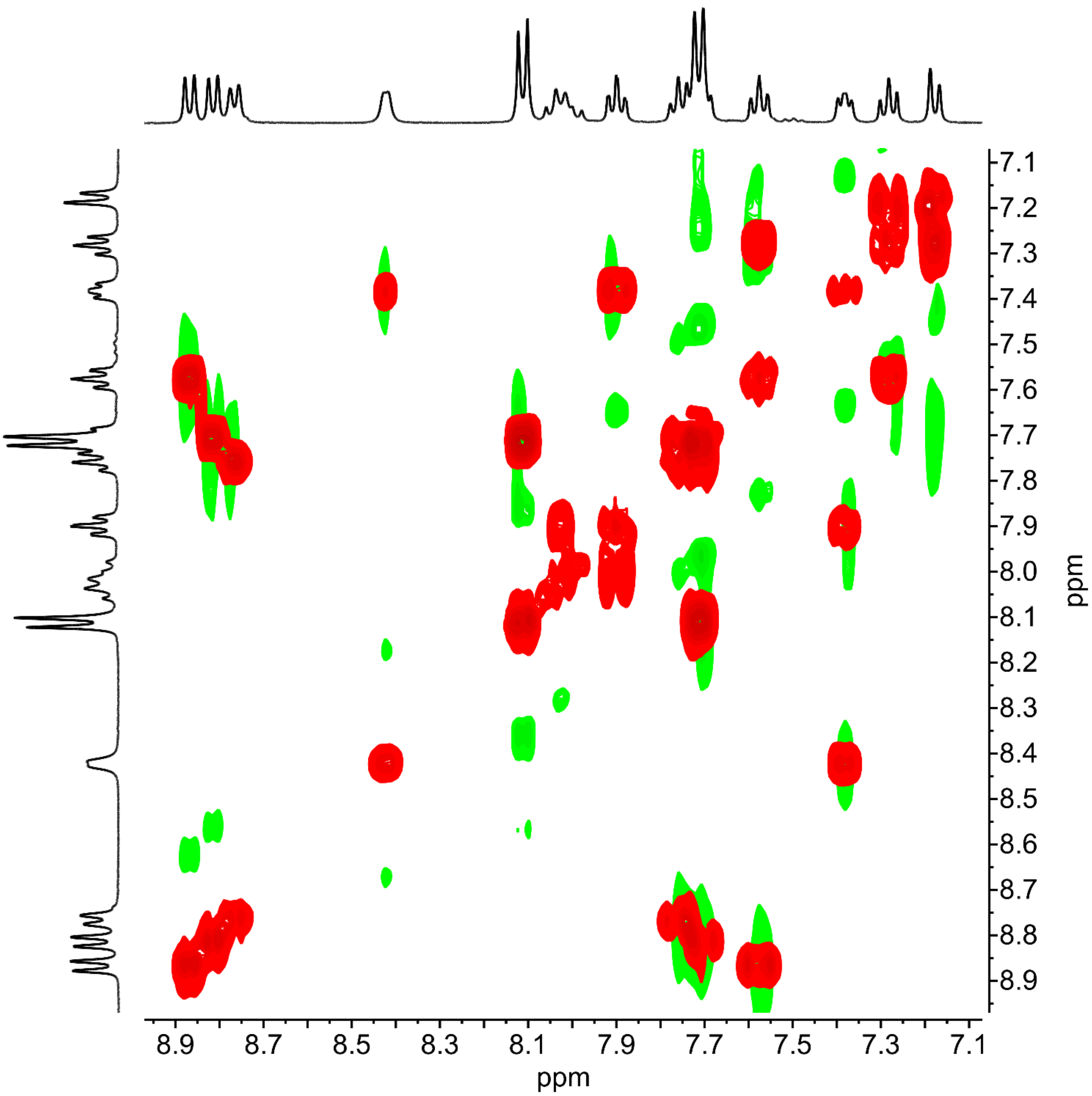

Figure S12. ^{1}H-^{1}H COSY and NOESY NMR spectra of **N^N2**, CD_3OD, 298 K. Red diagonal and crosspeaks are from COSY spectrum, green crosspeaks are from NOESY spectrum.

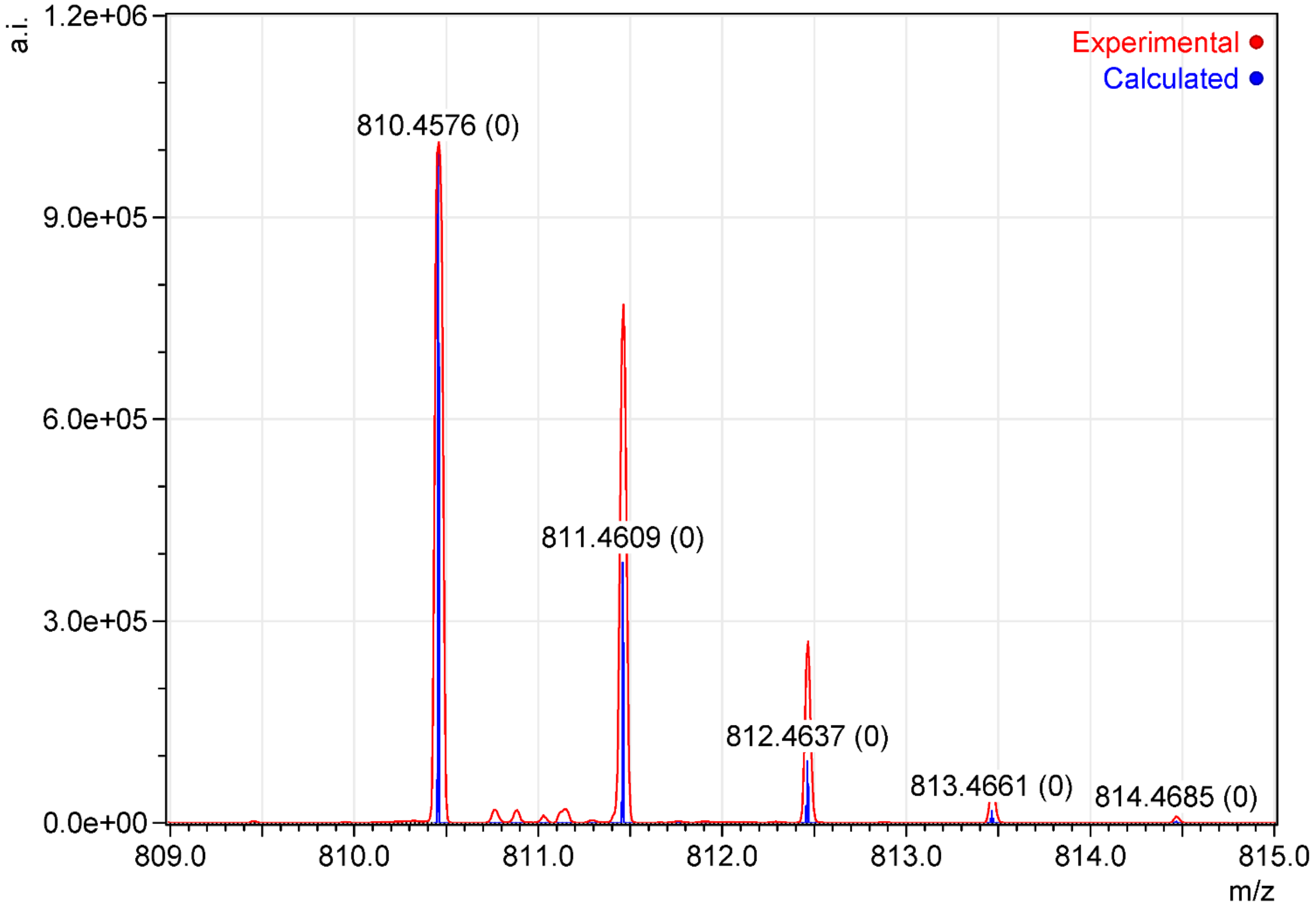

Figure S13. ESI$^+$ mass-spectrum of **NHBoc-4OEG** ([M+Na]$^+$ cation area), solvent – methanol.

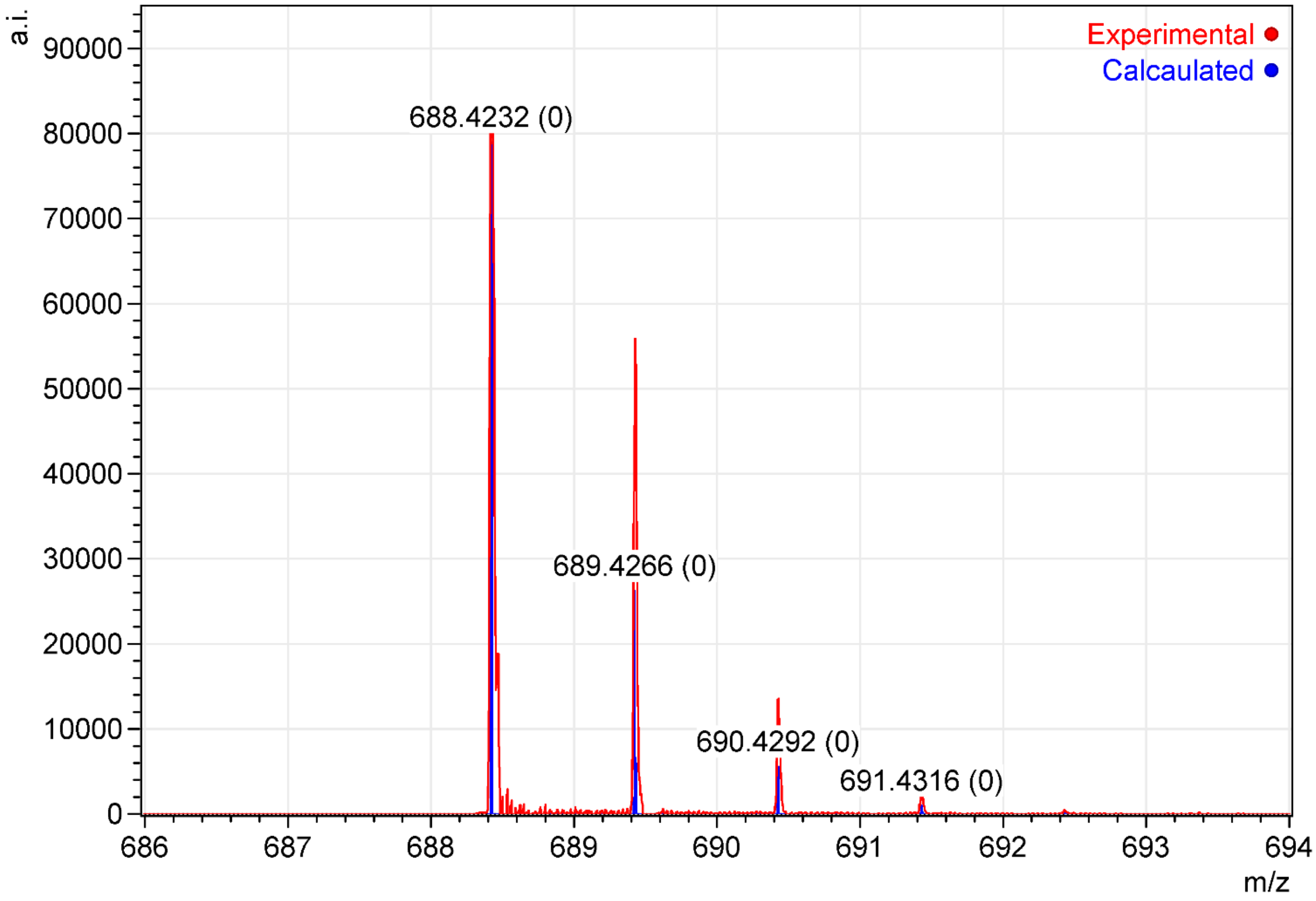

Figure S14. ESI$^+$ mass-spectrum of **NH_2-4OEG** ($[M+H]^+$ cation area), solvent – methanol.

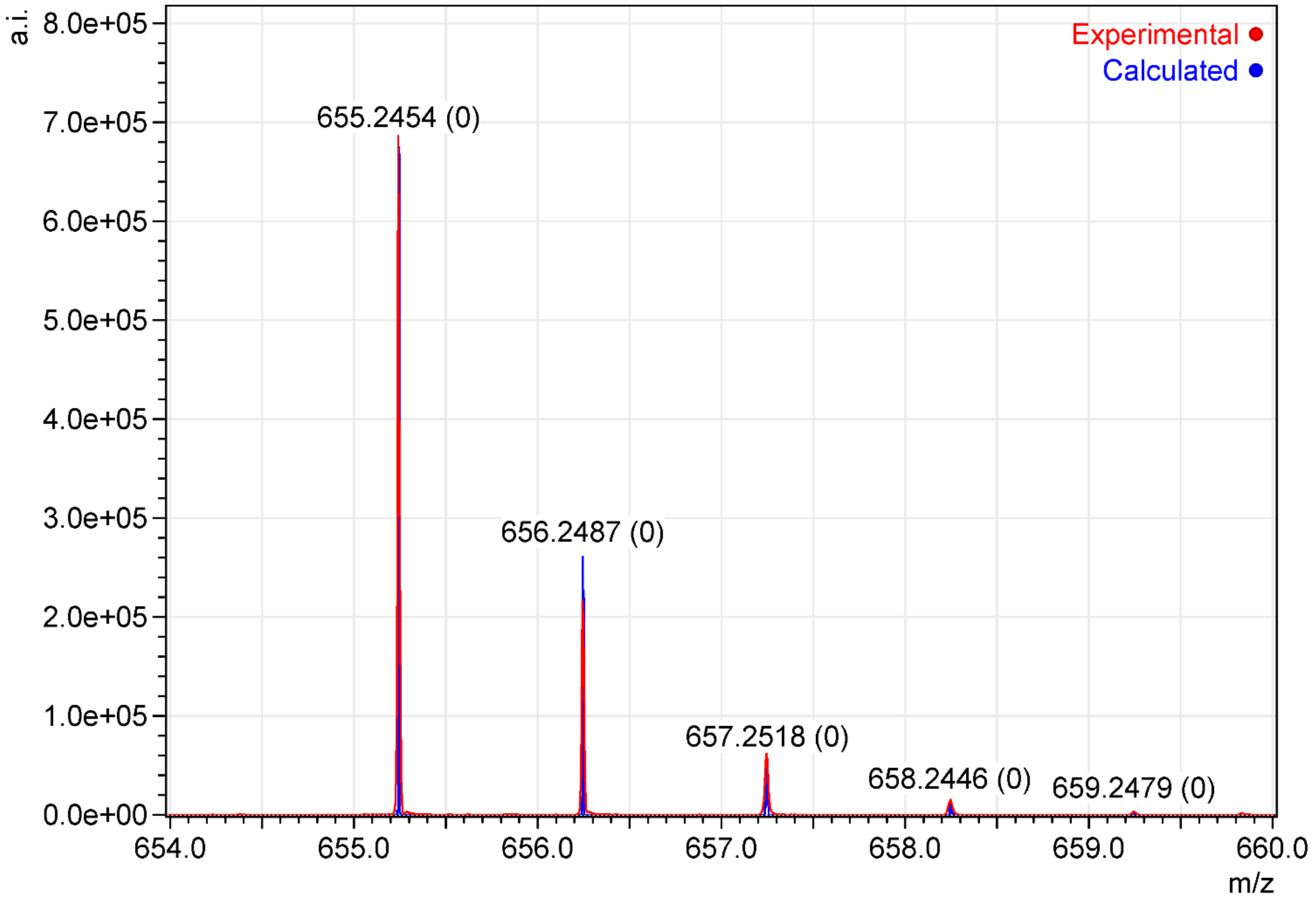

Figure S15. ESI$^+$ mass-spectrum of **N^C1** ($[M+Na]^+$ cation area), solvent – methanol.

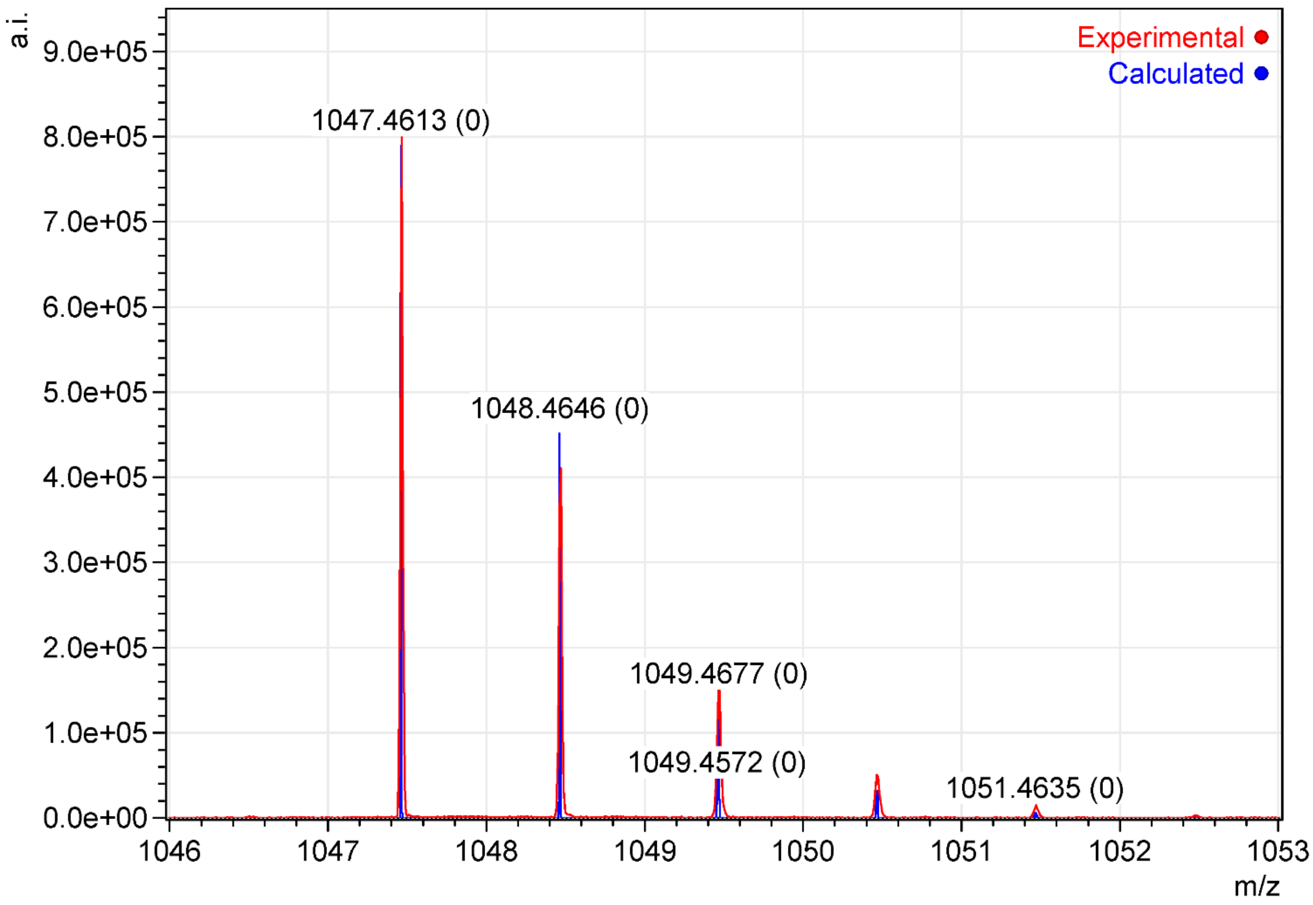

Figure S16. ESI$^+$ mass-spectrum of **N^C2** ([M+Na]$^+$ cation area), solvent – methanol.

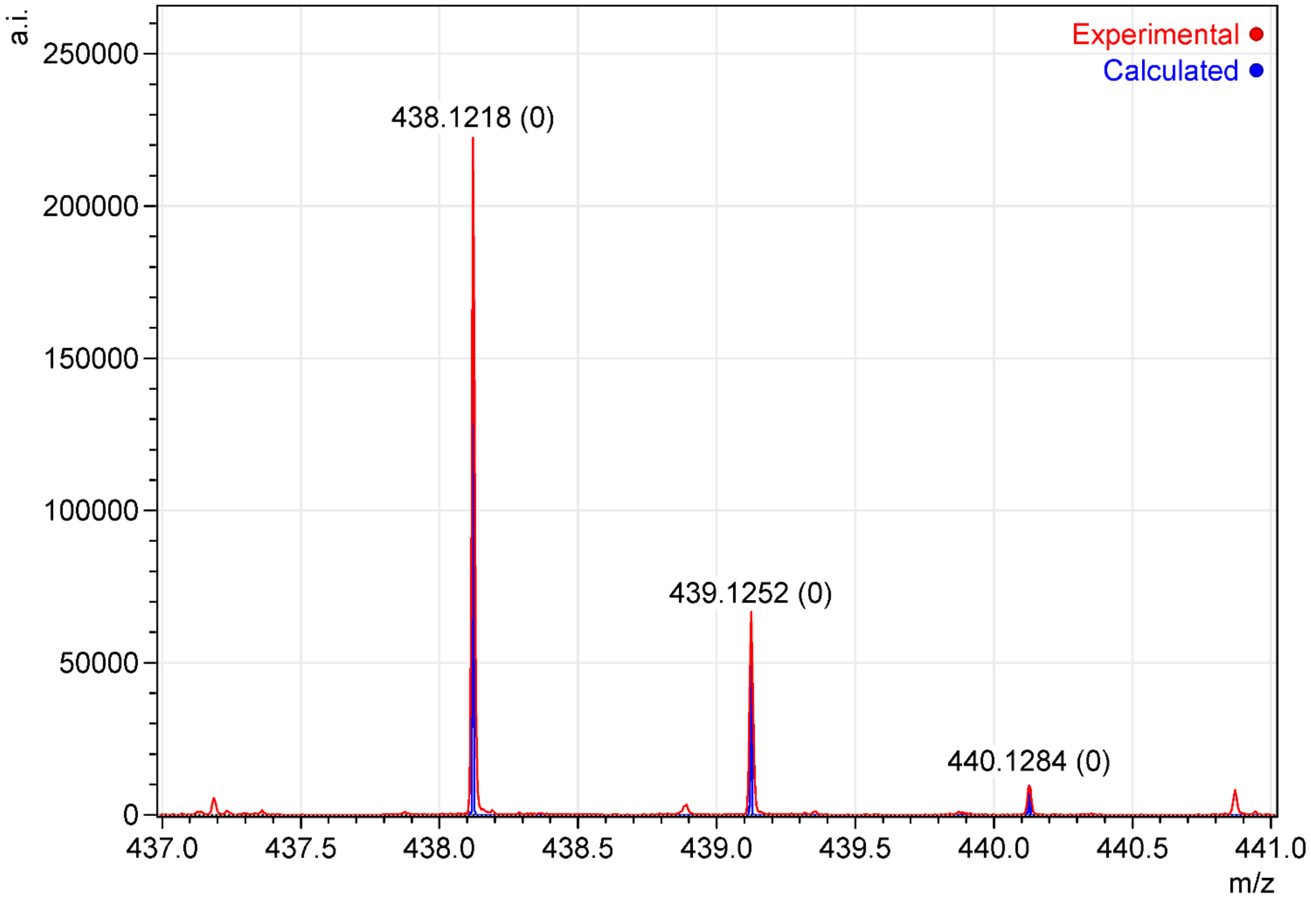

Figure S17. ESI$^+$ mass-spectrum of 4-(2-(pyridin-2-yl)-1H-phenanthro[9,10-d]imidazol-1-yl)benzoic acid ([M+Na]$^+$ cation area), solvent – methanol.

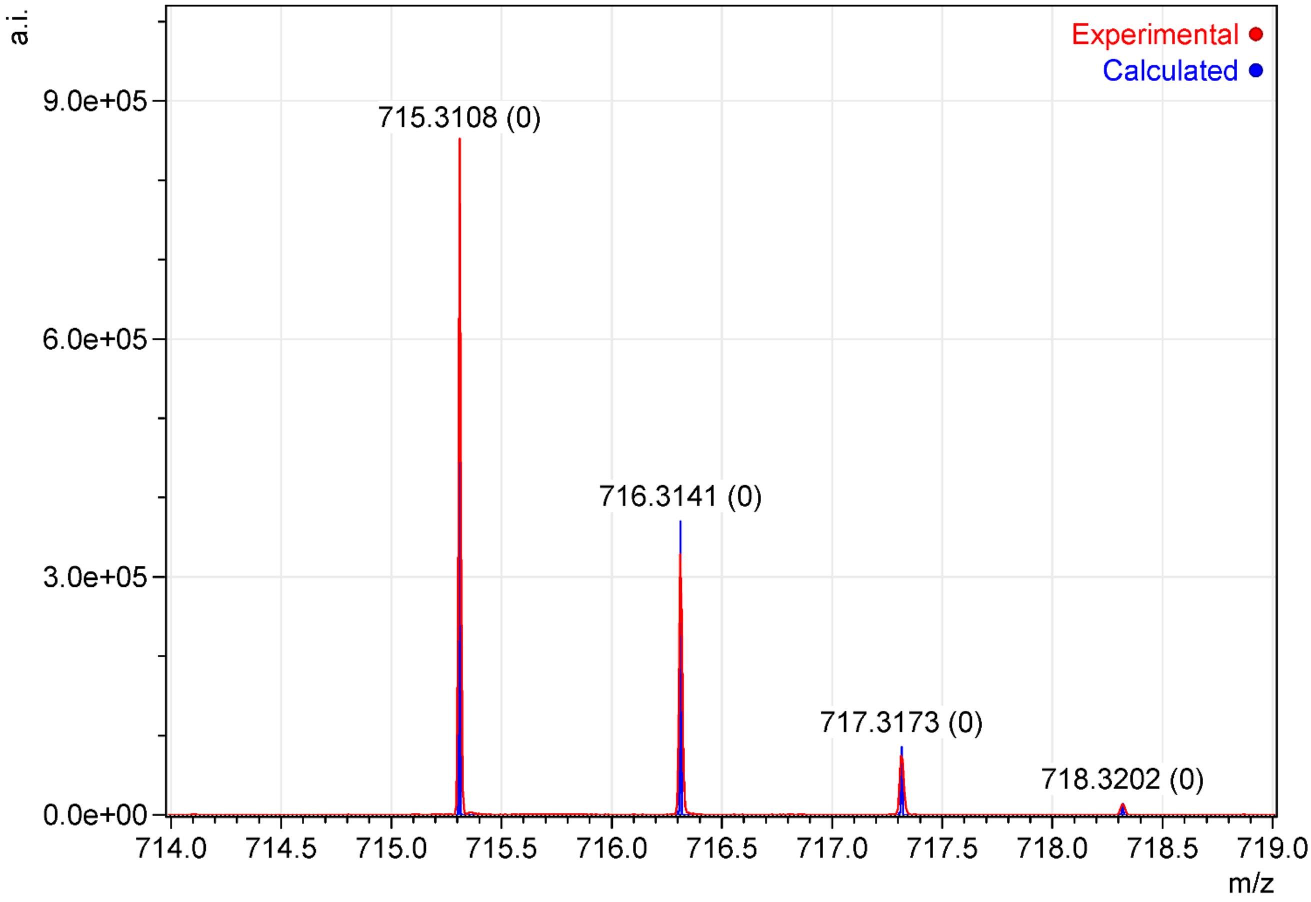

Figure S18. ESI$^+$ mass-spectrum of **N^N1** ($[M+Na]^+$ cation area), solvent – methanol.

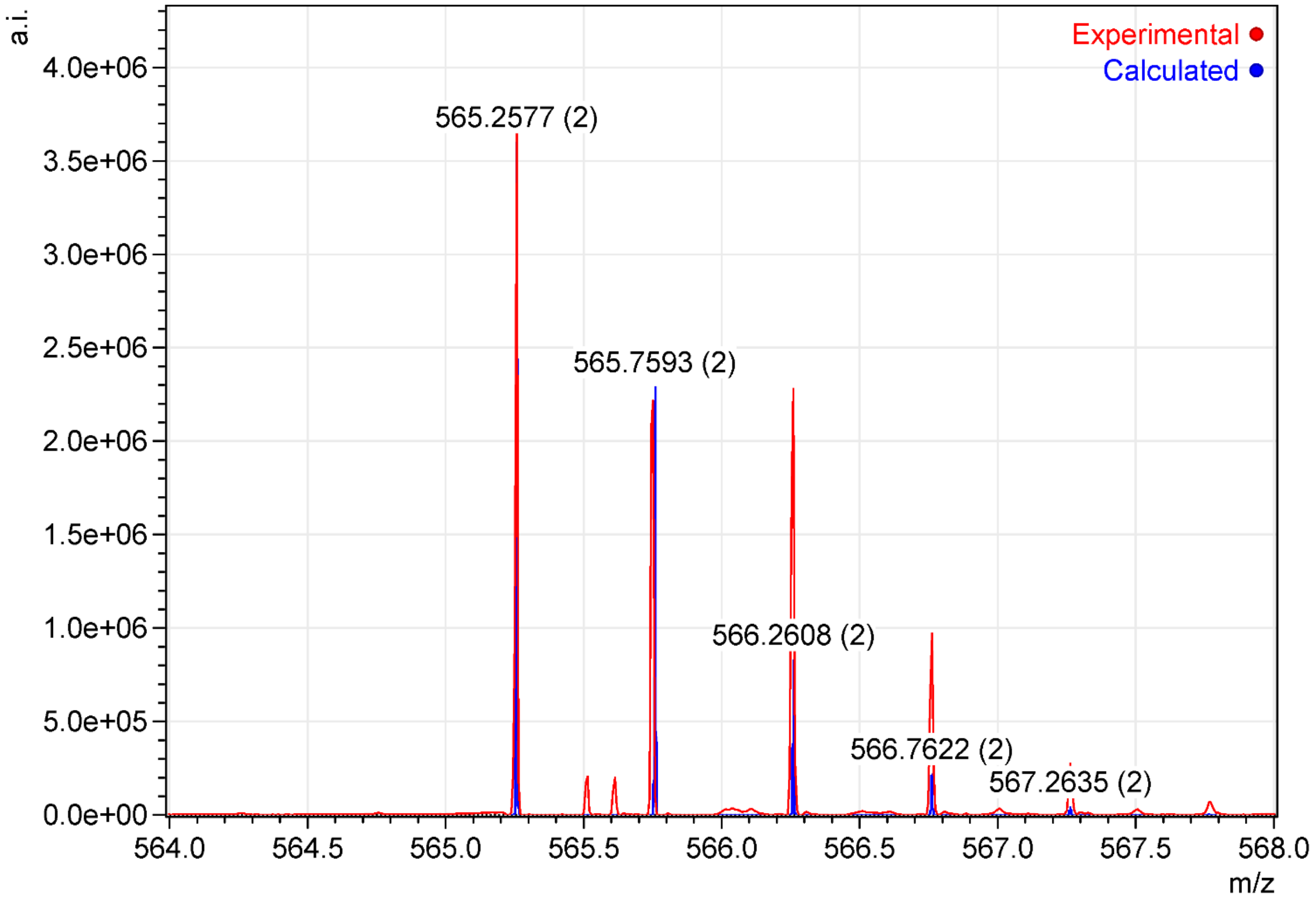

Figure S19. ESI+ mass-spectrum of **N^N2** ($[M+2Na]^{2+}$ cation area), solvent – methanol.

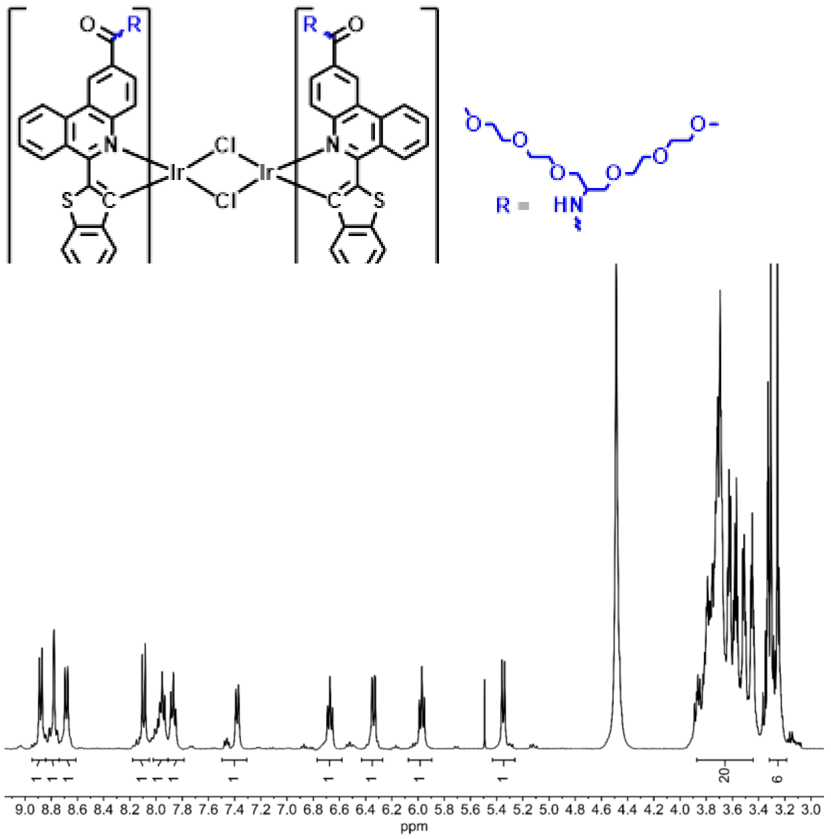

Figure S20. ^{1}H NMR spectrum of **D1**, CD_3OD, 298 K.

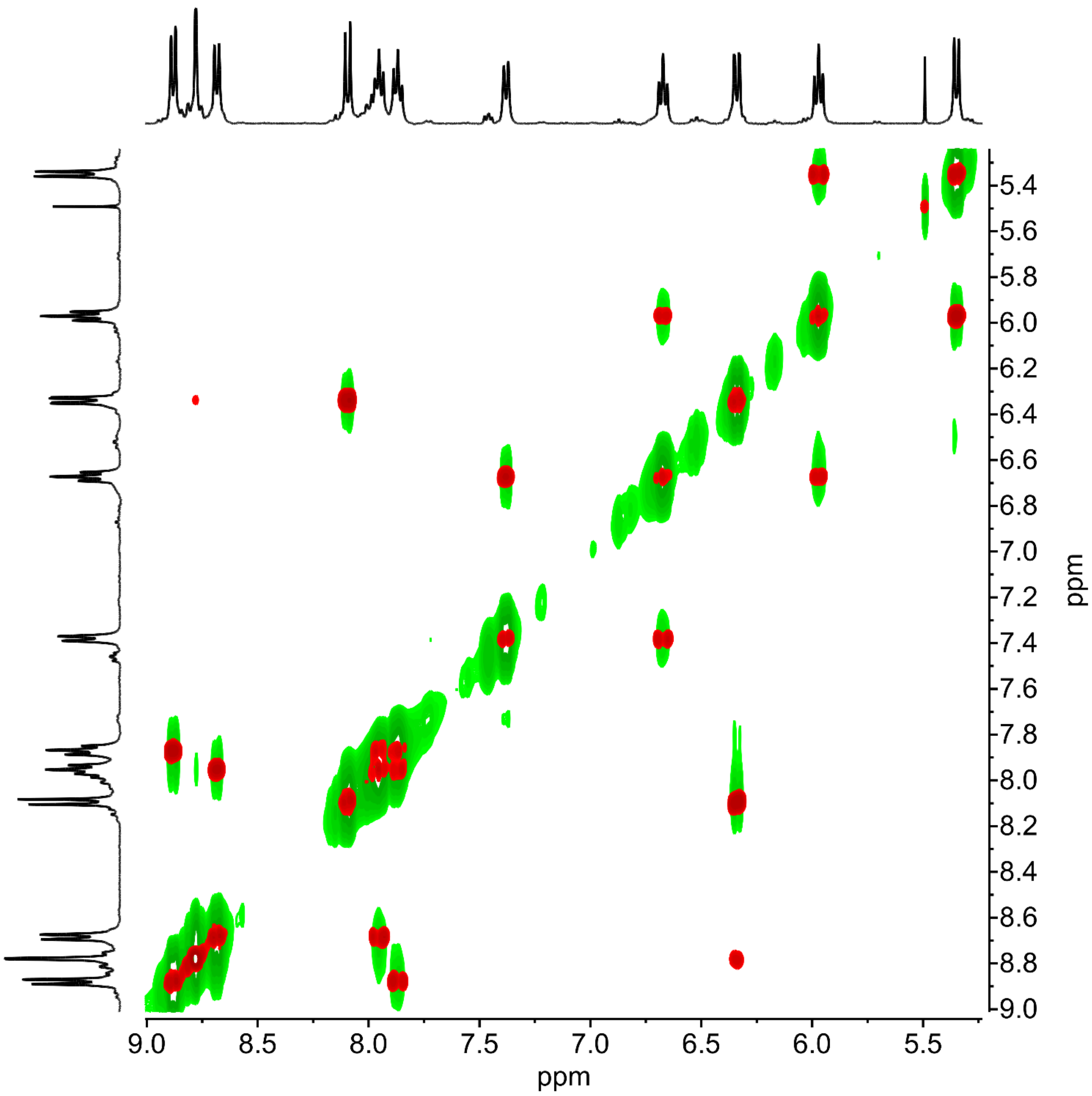

Figure S21. ^1H-^1H COSY and NOESY NMR spectra of **D1**, CD_3OD, 298 K. Red diagonal and crosspeaks are from COSY spectrum, green crosspeaks are from NOESY spectrum.

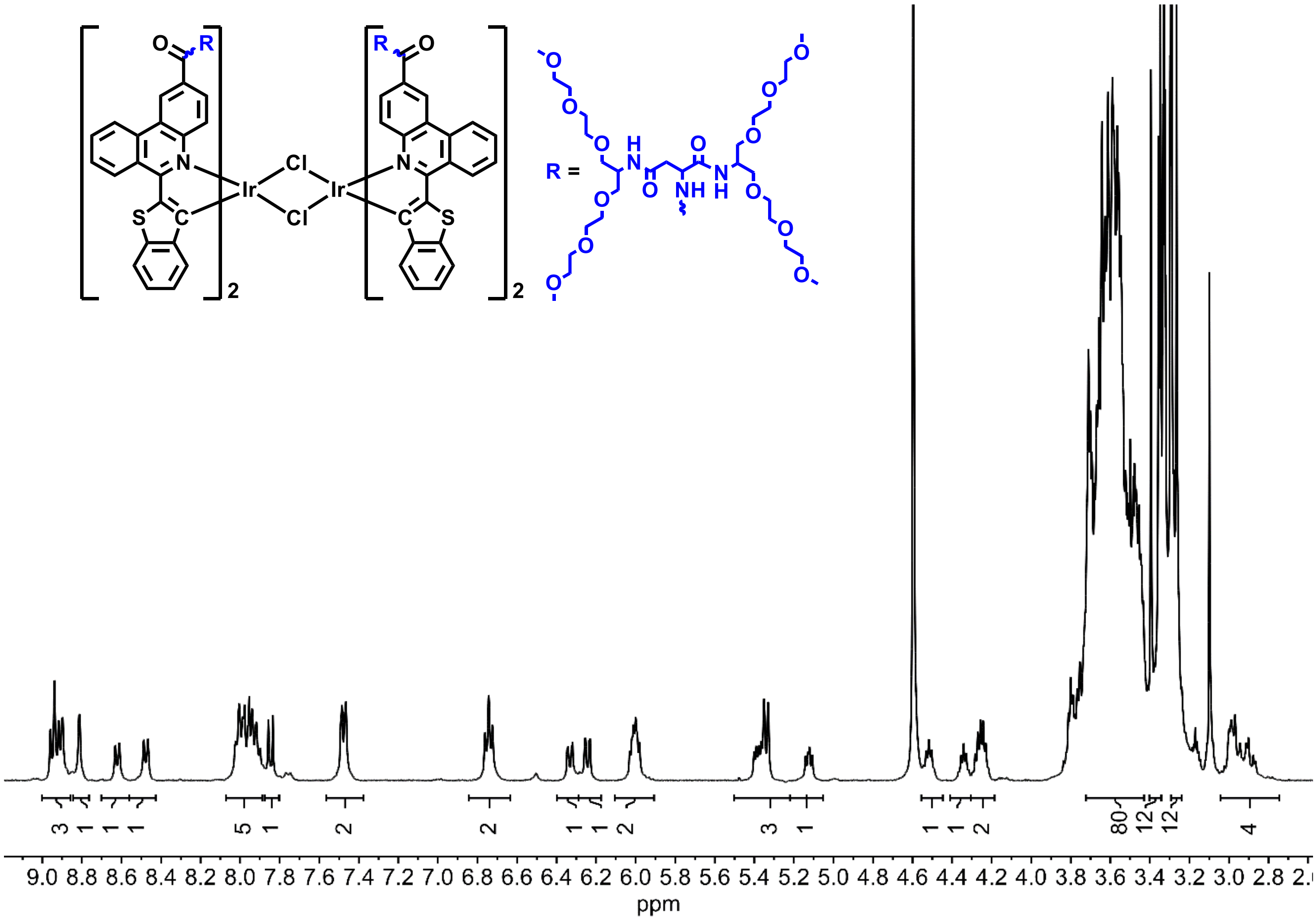

Figure S22. ^{1}H NMR spectrum of **D2**, CD_3OD, 323 K.

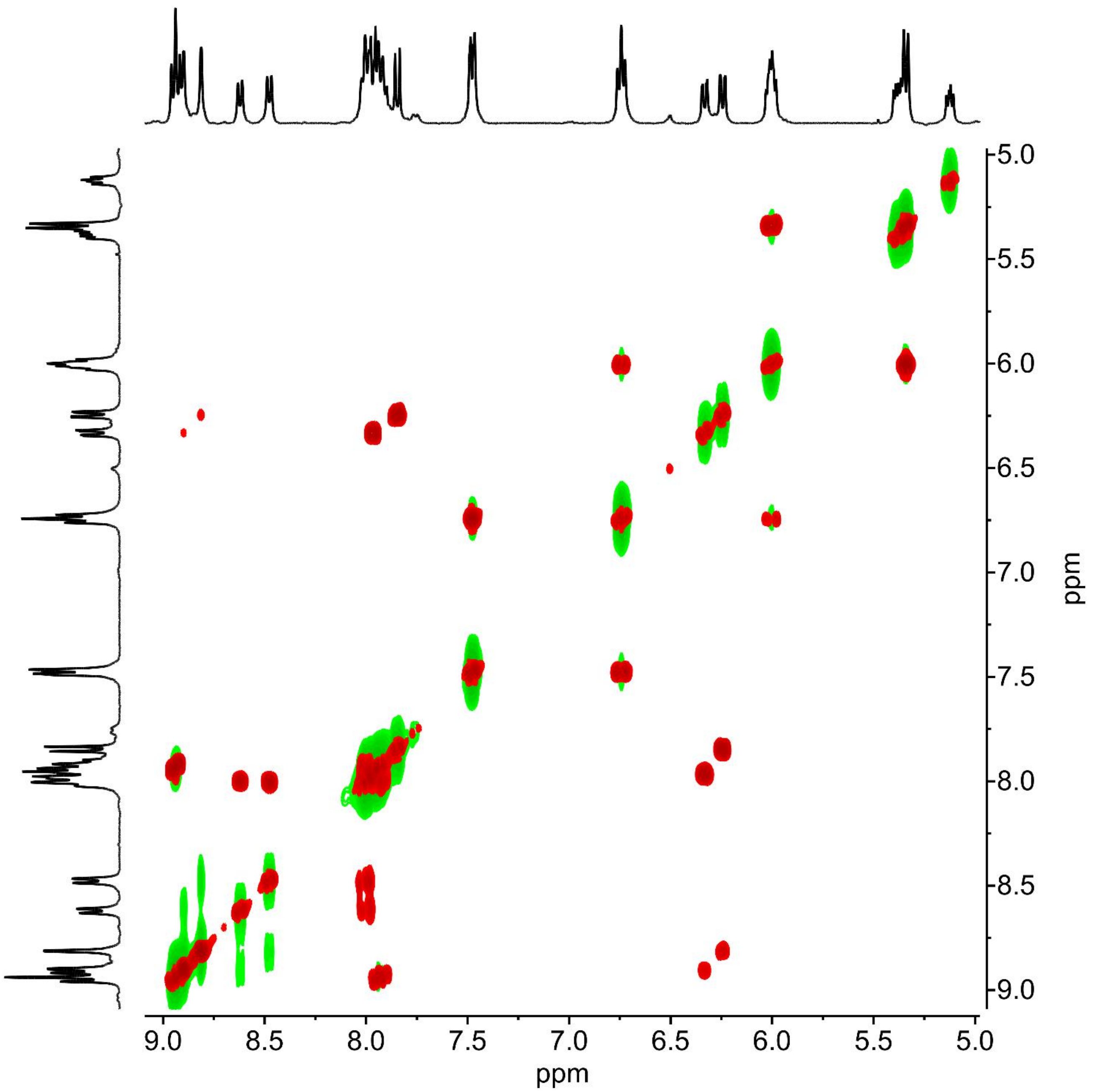

Figure S23. ^{1}H-^{1}H COSY and NOESY NMR spectra of **D2**, CD_3OD, 323 K. Red diagonal and crosspeaks are from COSY spectrum, green crosspeaks are from NOESY spectrum.

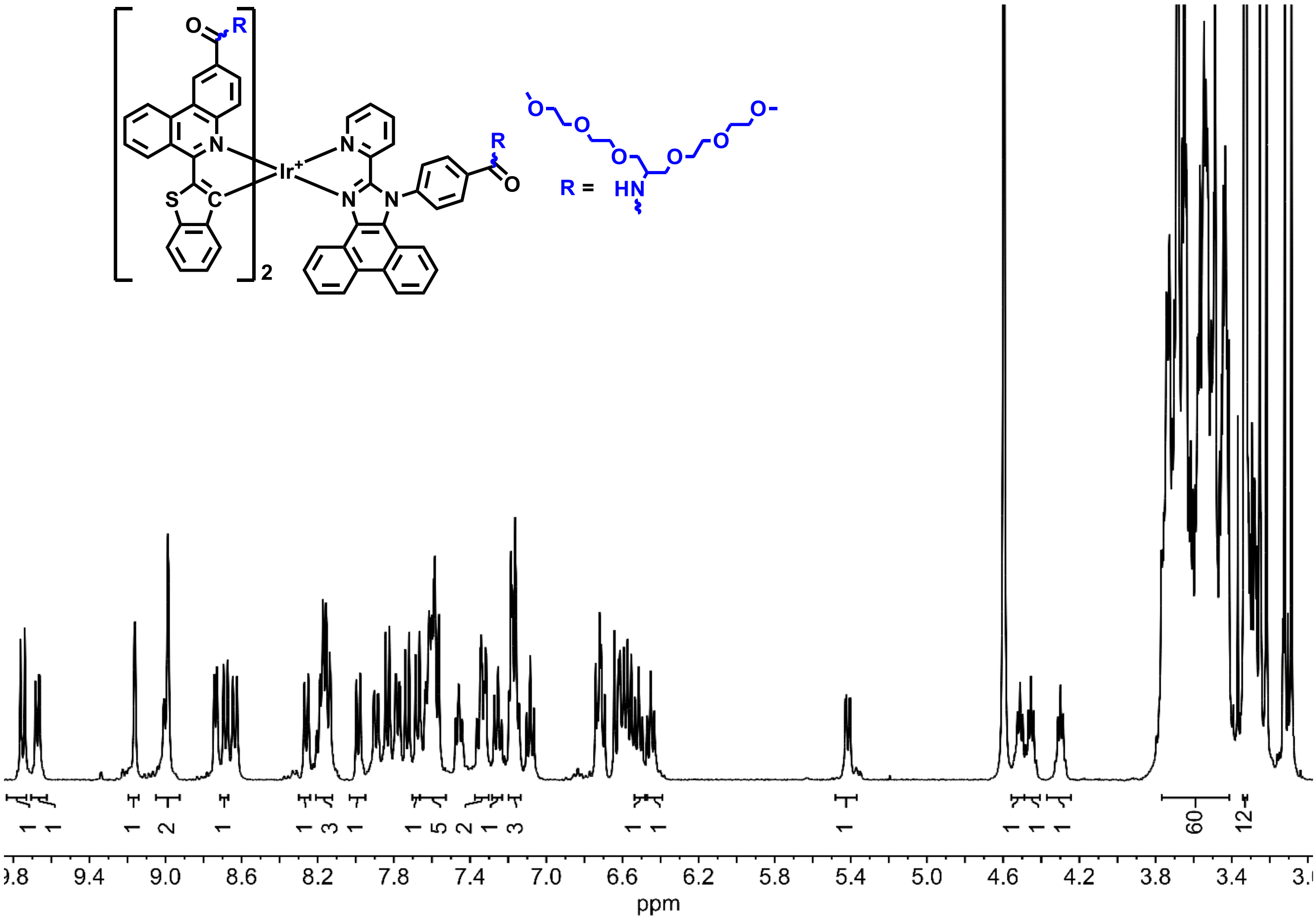

Figure S24. ^{1}H NMR spectrum of **Ir1**, CD_3OD, 323 K.

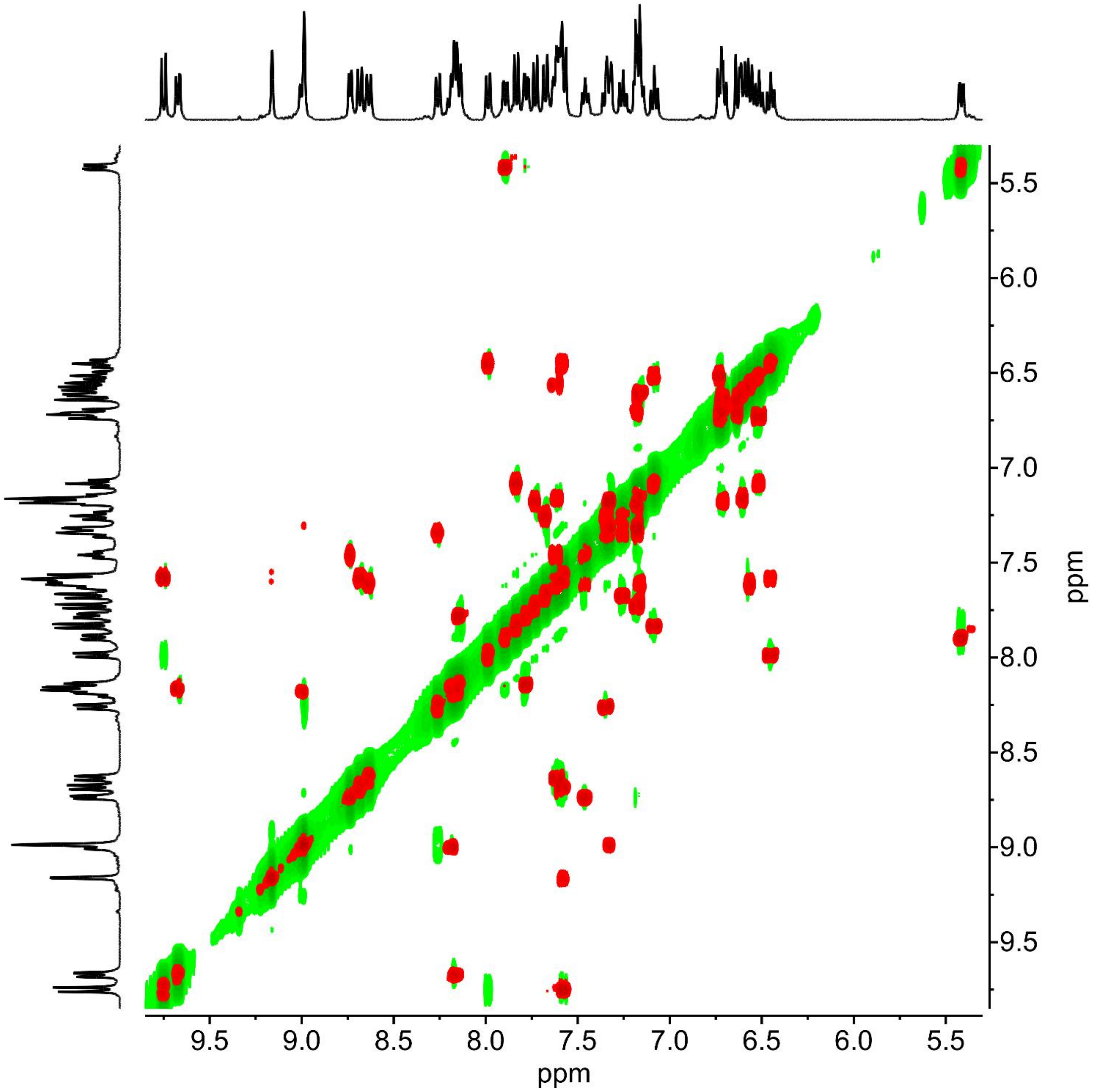

Figure S25. ^1H-^1H COSY and NOESY NMR spectra of **Ir1**, CD_3OD, 323 K. Red diagonal and crosspeaks are from COSY spectrum, green crosspeaks are from NOESY spectrum.

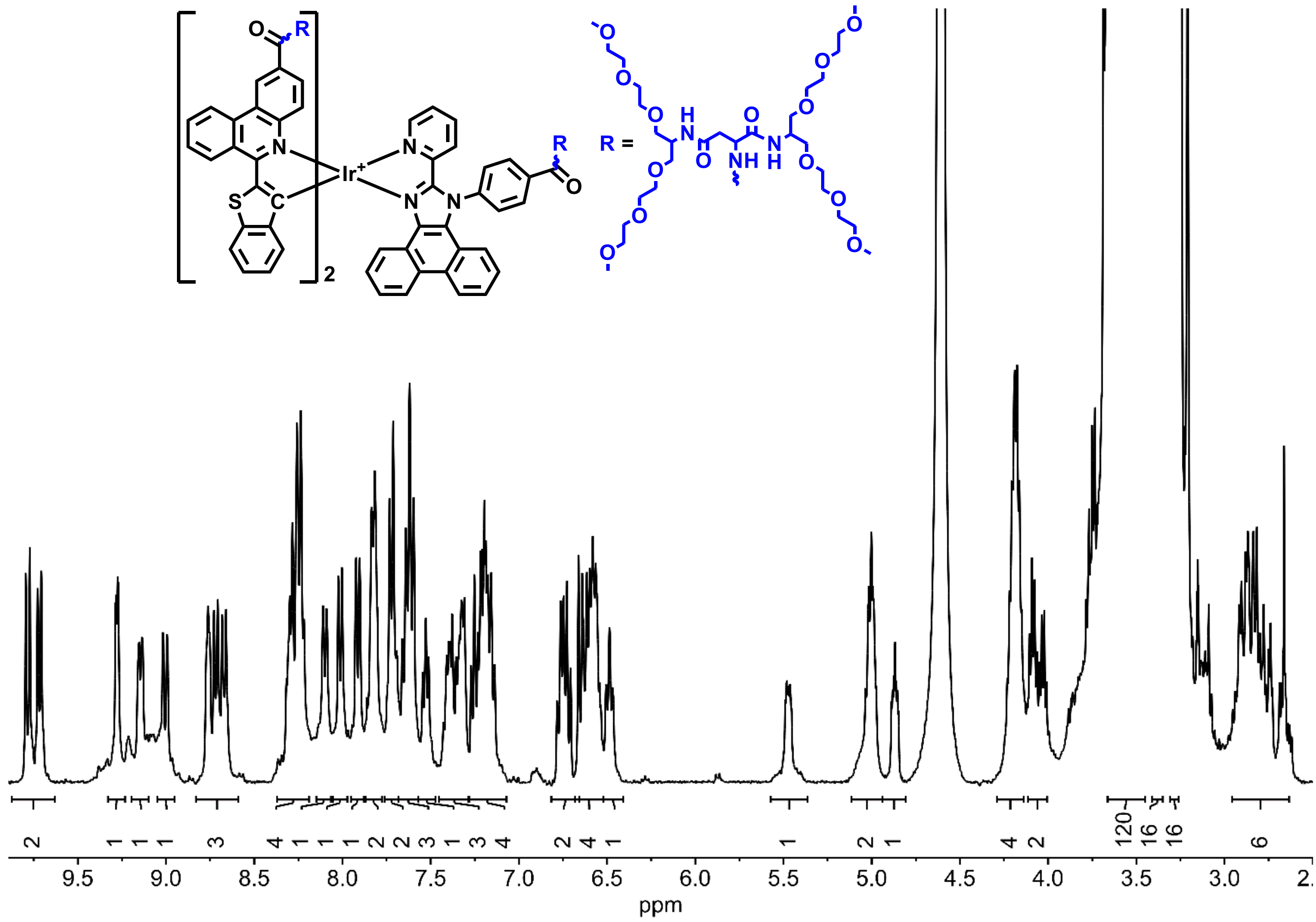

Figure S26. ^{1}H NMR spectrum of **Ir2**, CD_3OD, 323 K.

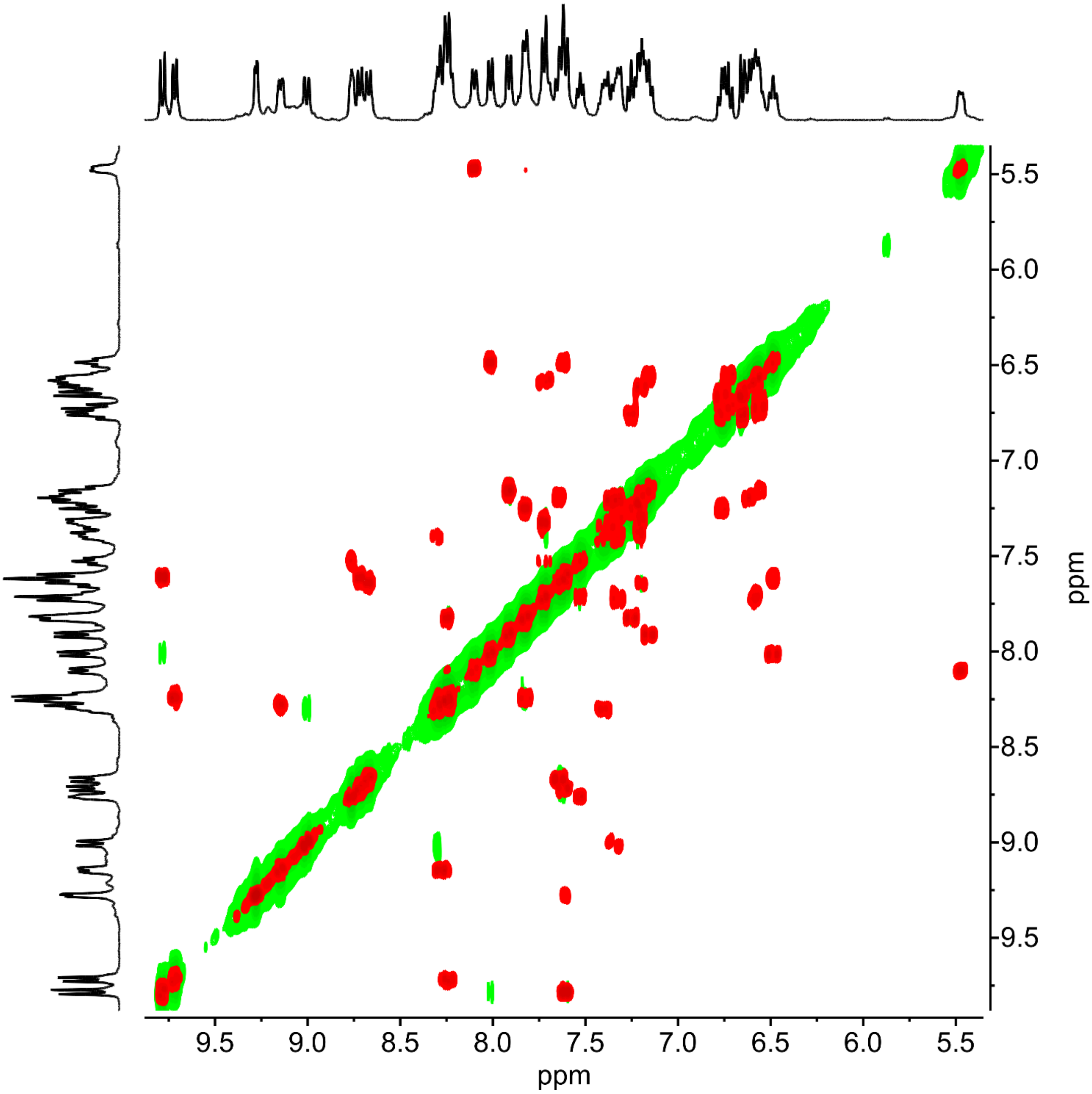

Figure S27. ^1H-^1H COSY and NOESY NMR spectra of **Ir2**, CD_3OD, 323 K. Red diagonal and crosspeaks are from COSY spectrum, green crosspeaks are from NOESY spectrum.

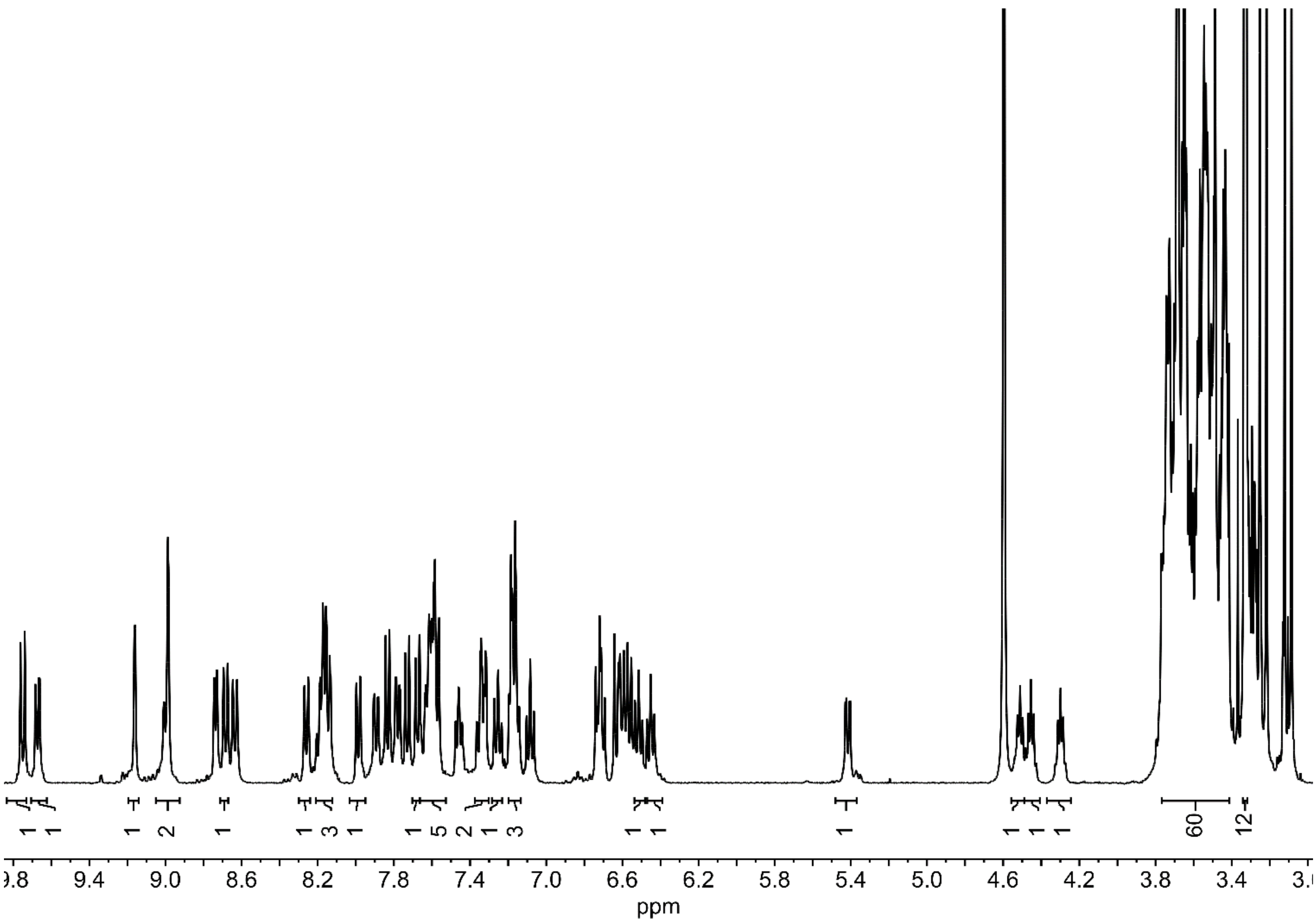

Figure S28. ^{1}H NMR spectrum of **Ir1a**, CD_3OD, 323 K.

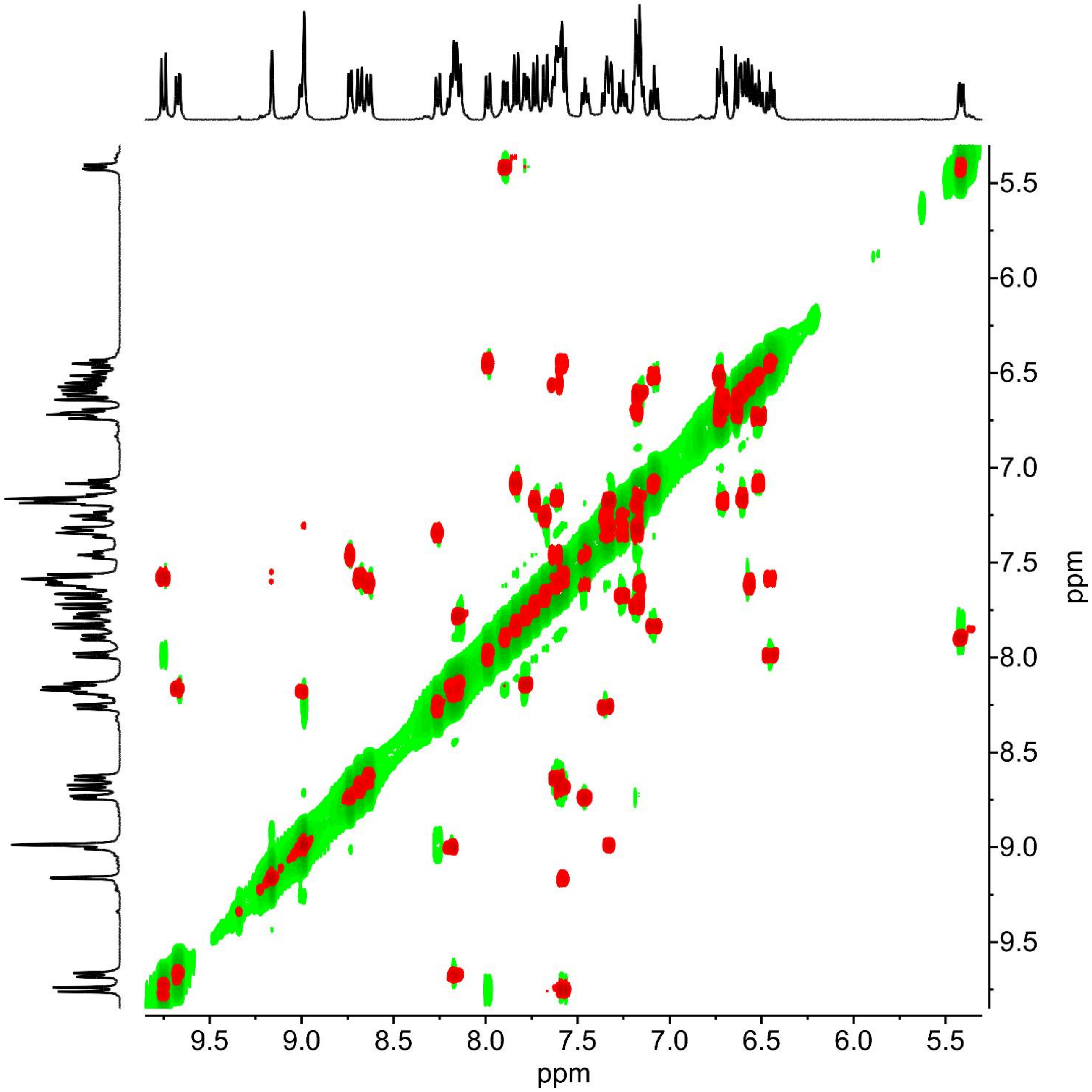

Figure S29. ^{1}H-^{1}H COSY and NOESY NMR spectra of **Ir1a**, CD_3OD, 323 K. Red diagonal and crosspeaks are from COSY spectrum, green crosspeaks are from NOESY spectrum.

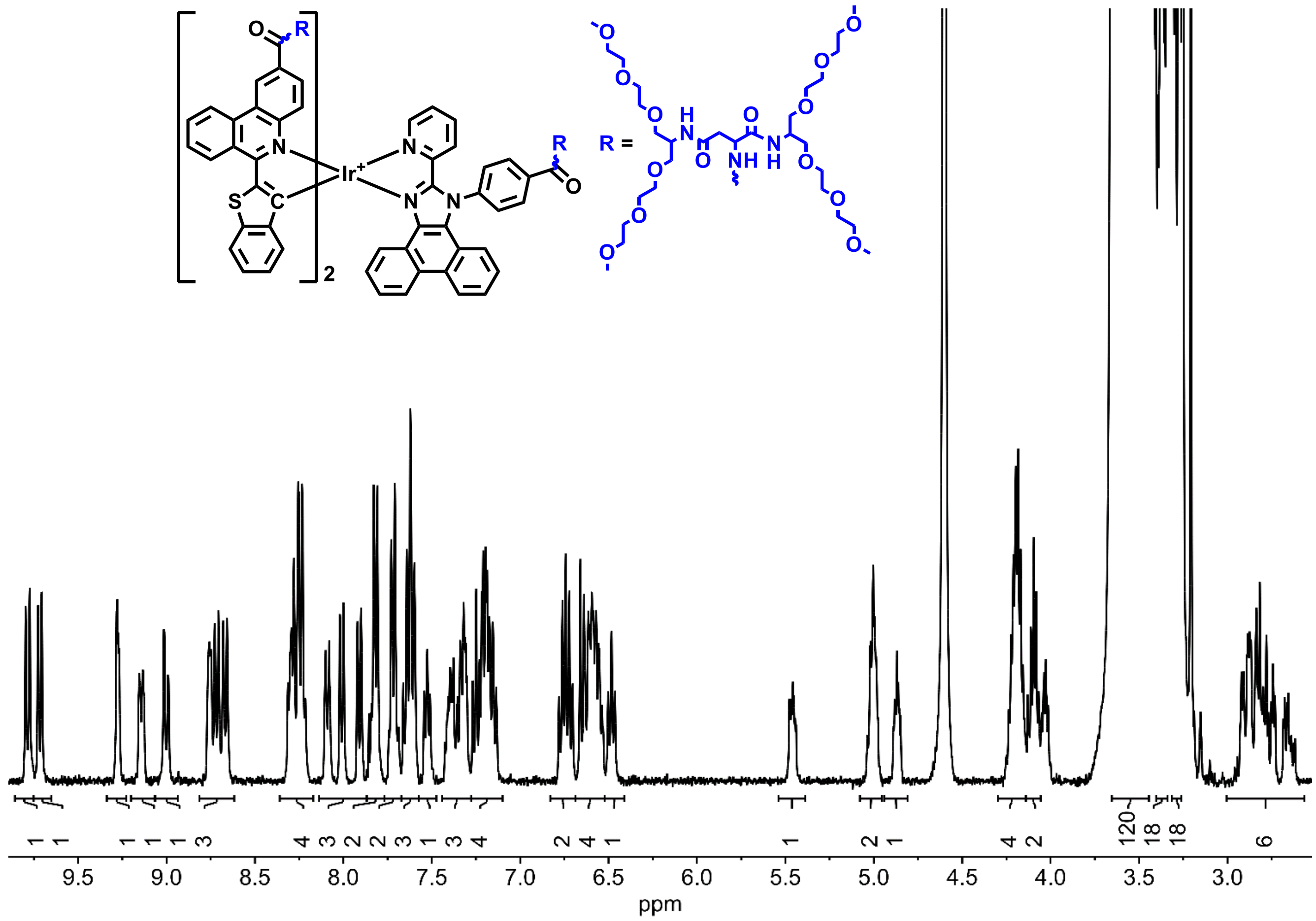

Figure S30. ^{1}H NMR spectrum of **Ir2a**, CD_3OD, 323 K.

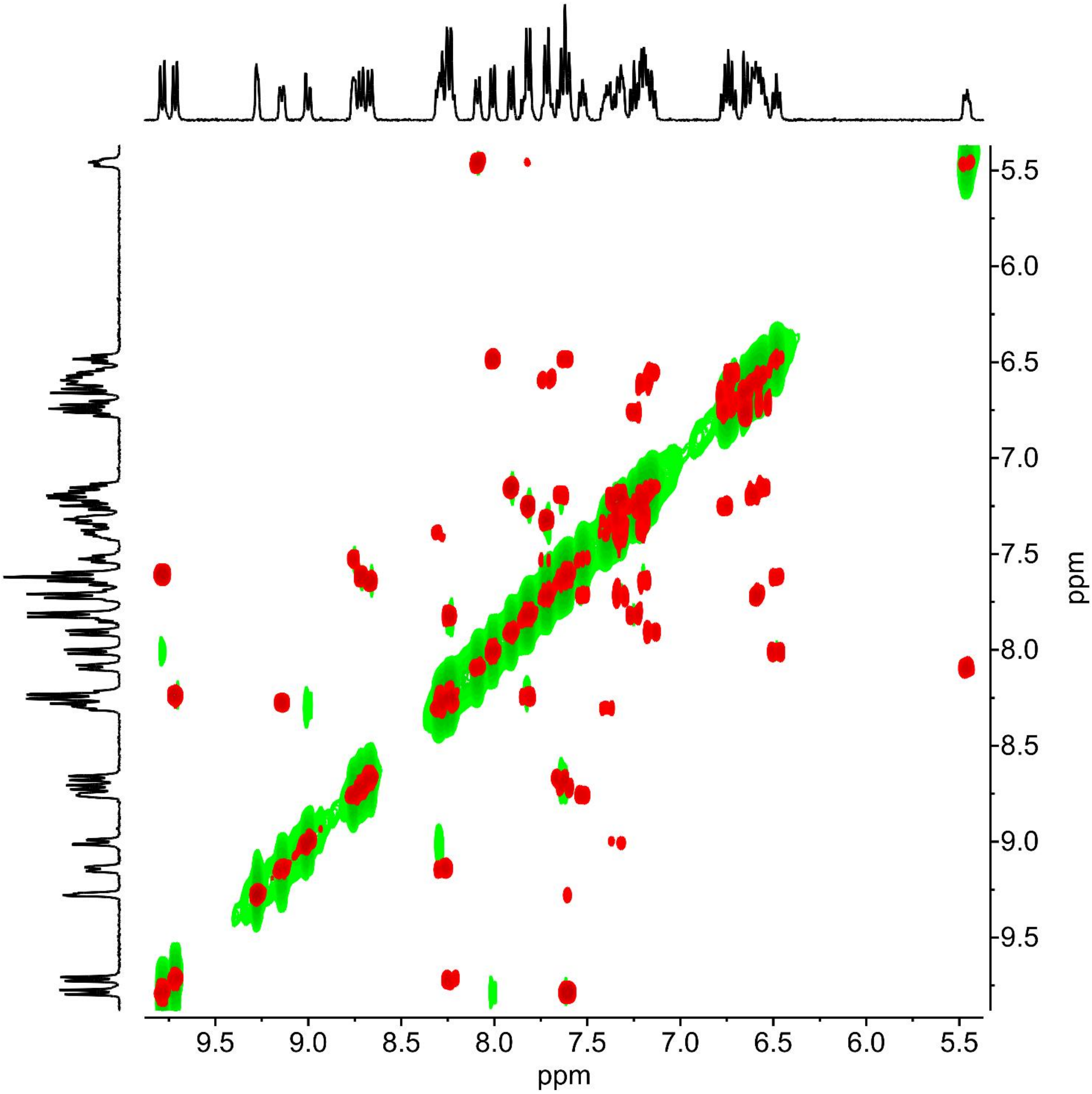

Figure S31. ^{1}H-^{1}H COSY and NOESY NMR spectra of **Ir2a**, CD_3OD, 323 K. Red diagonal and crosspeaks are from COSY spectrum, green crosspeaks are from NOESY spectrum.

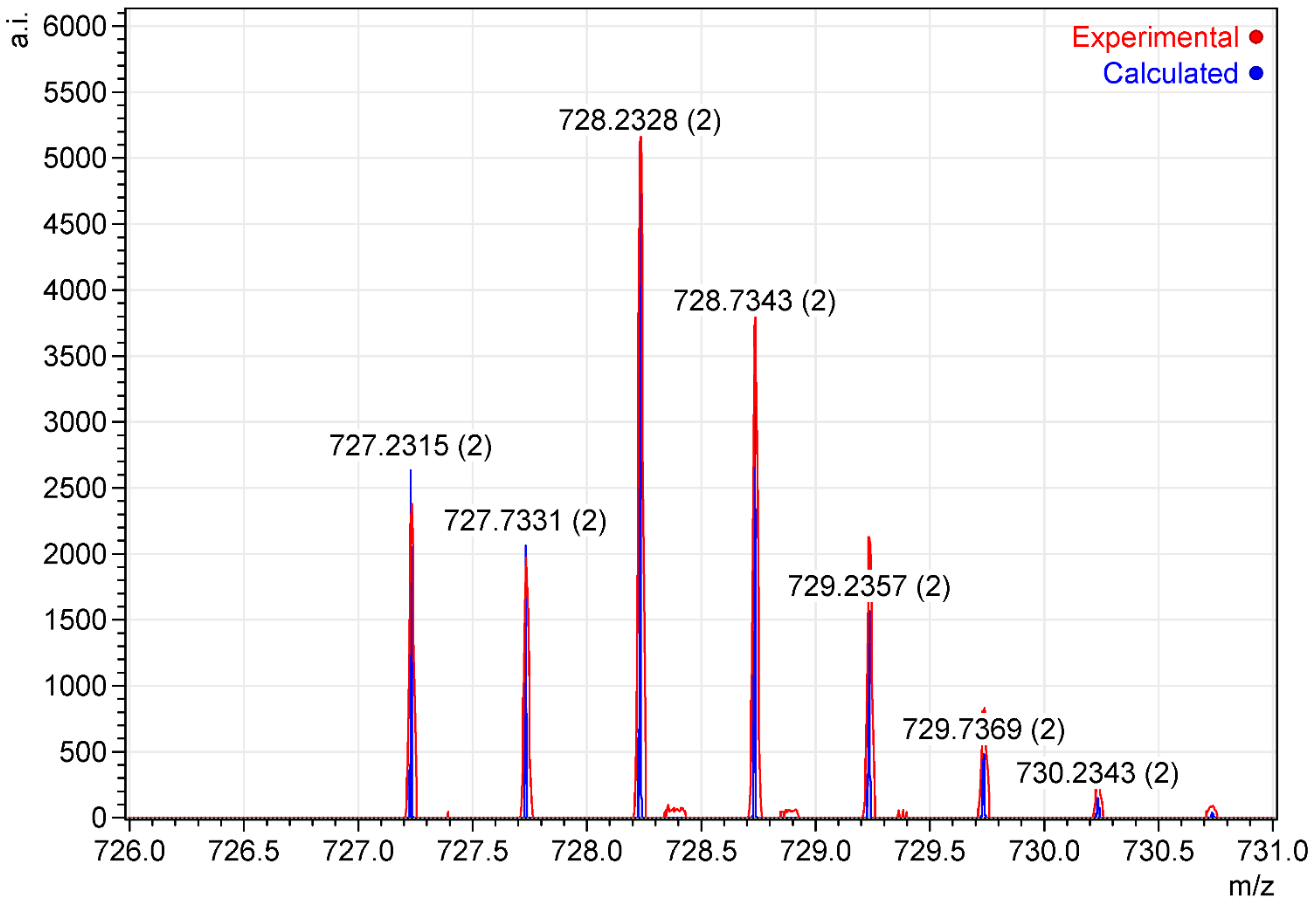

Figure S32. ESI$^+$ mass-spectrum of **D1** ([Ir(**N^C1**)$_2$+H]$^{2+}$ cation area), solvent – methanol.

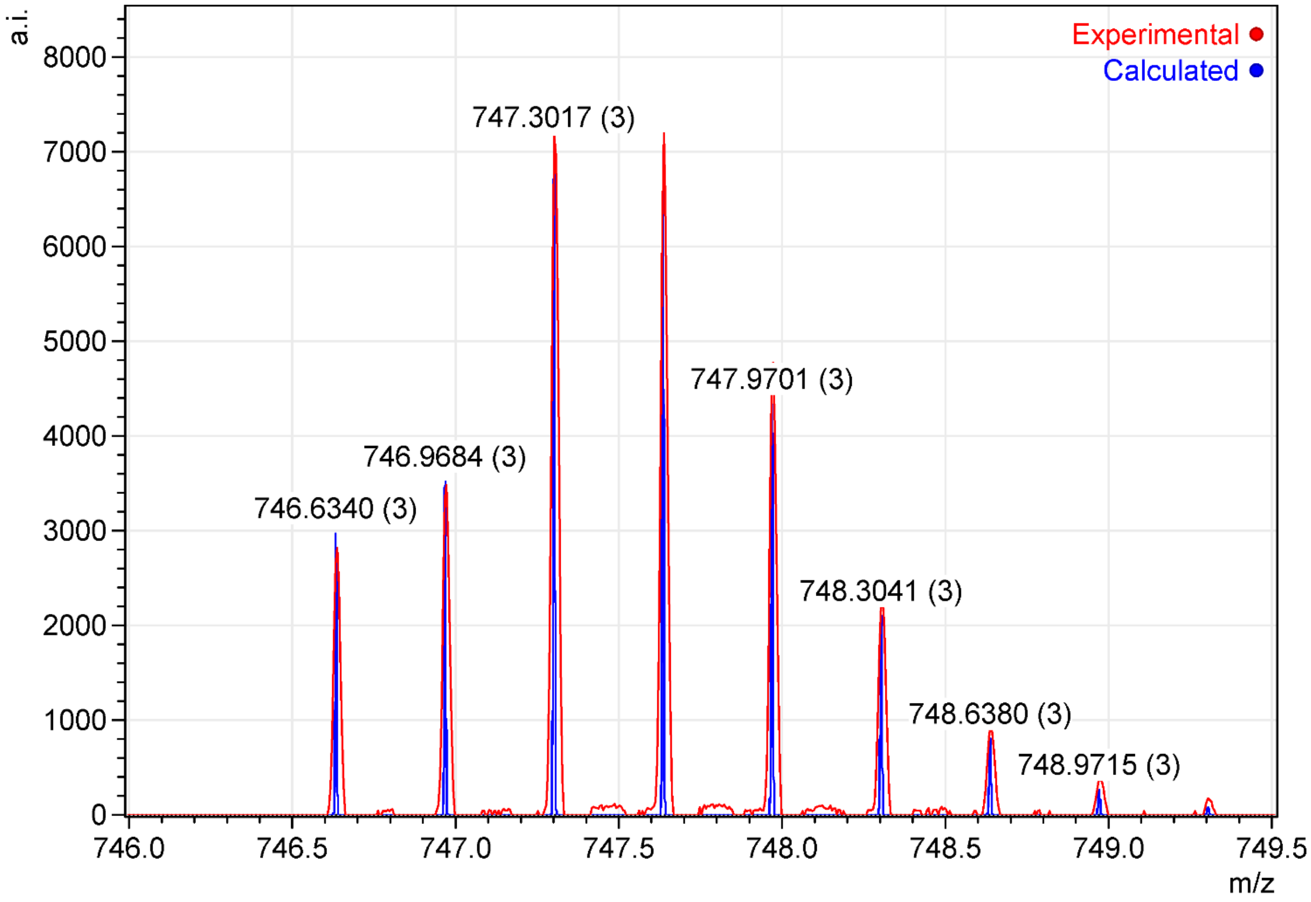

Figure S33. ESI^{+} mass-spectrum of **D2** ($[Ir($**N^C2**$)_2+2H]^{3+}$ cation area), solvent – methanol.

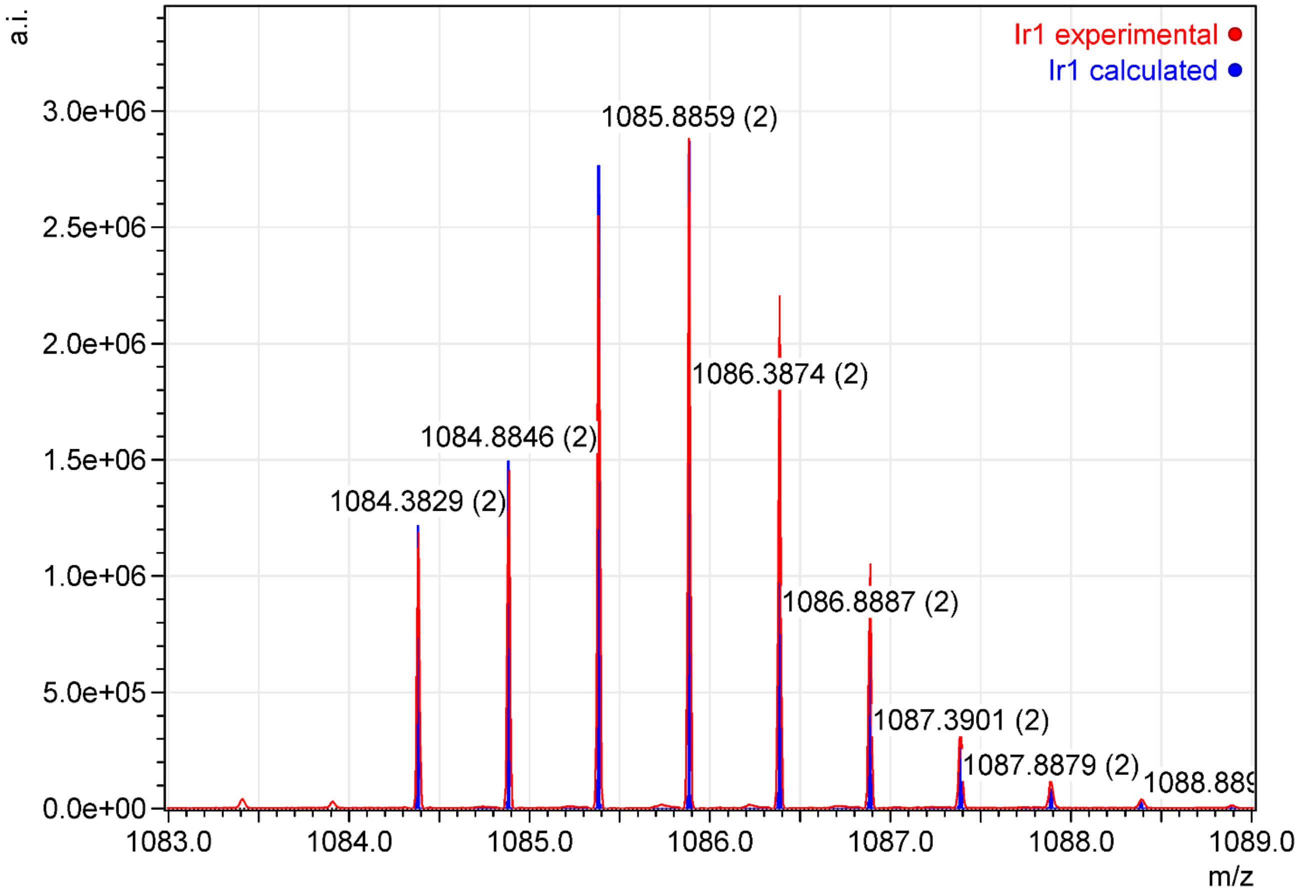

Figure S34. ESI+ mass-spectrum of **Ir1** ($[M+Na]^{2+}$ cation area), solvent – methanol.

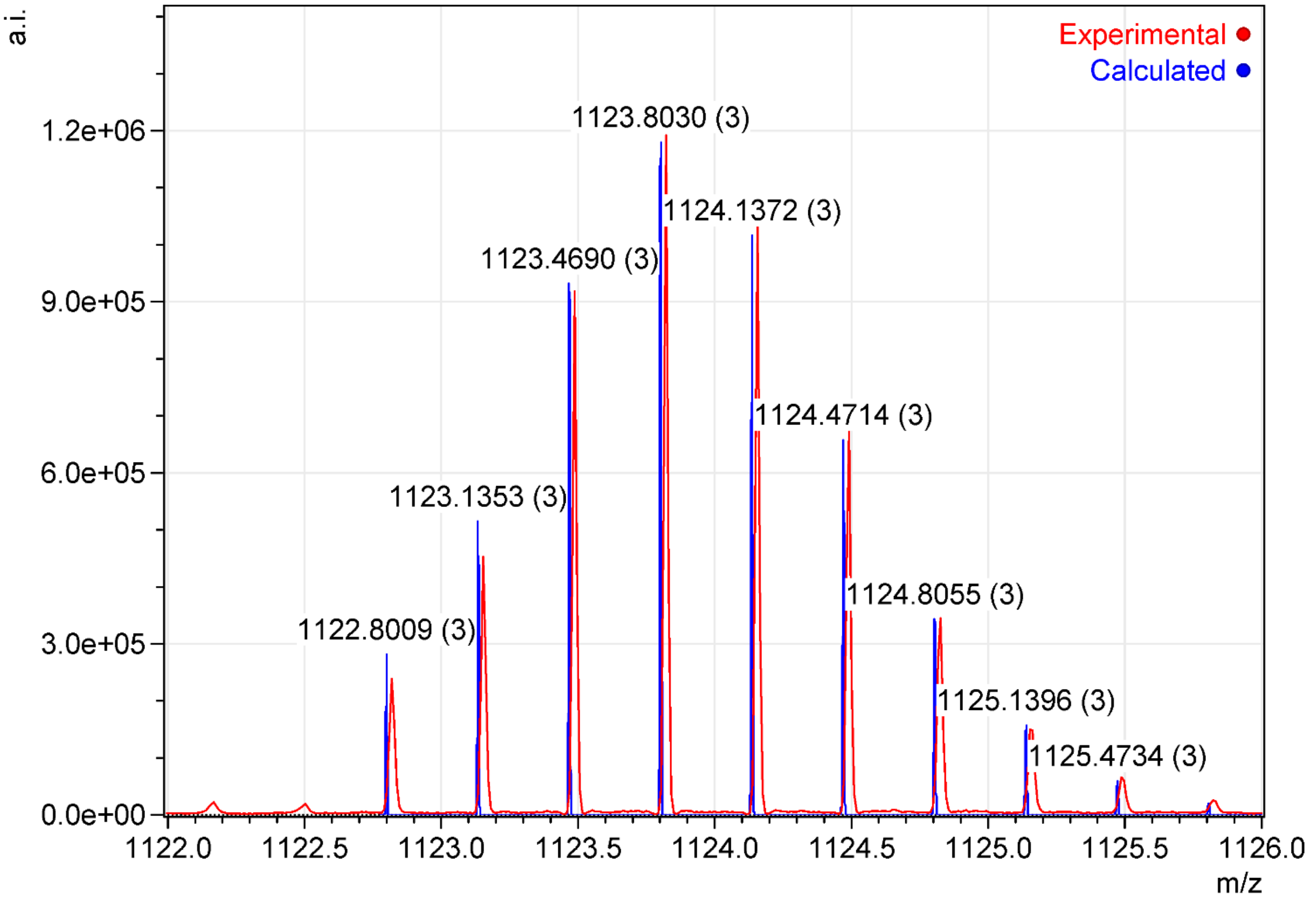

Figure S35. ESI$^+$ mass-spectrum of **Ir2** ($[M+2Na]^{3+}$ cation area), solvent – methanol.

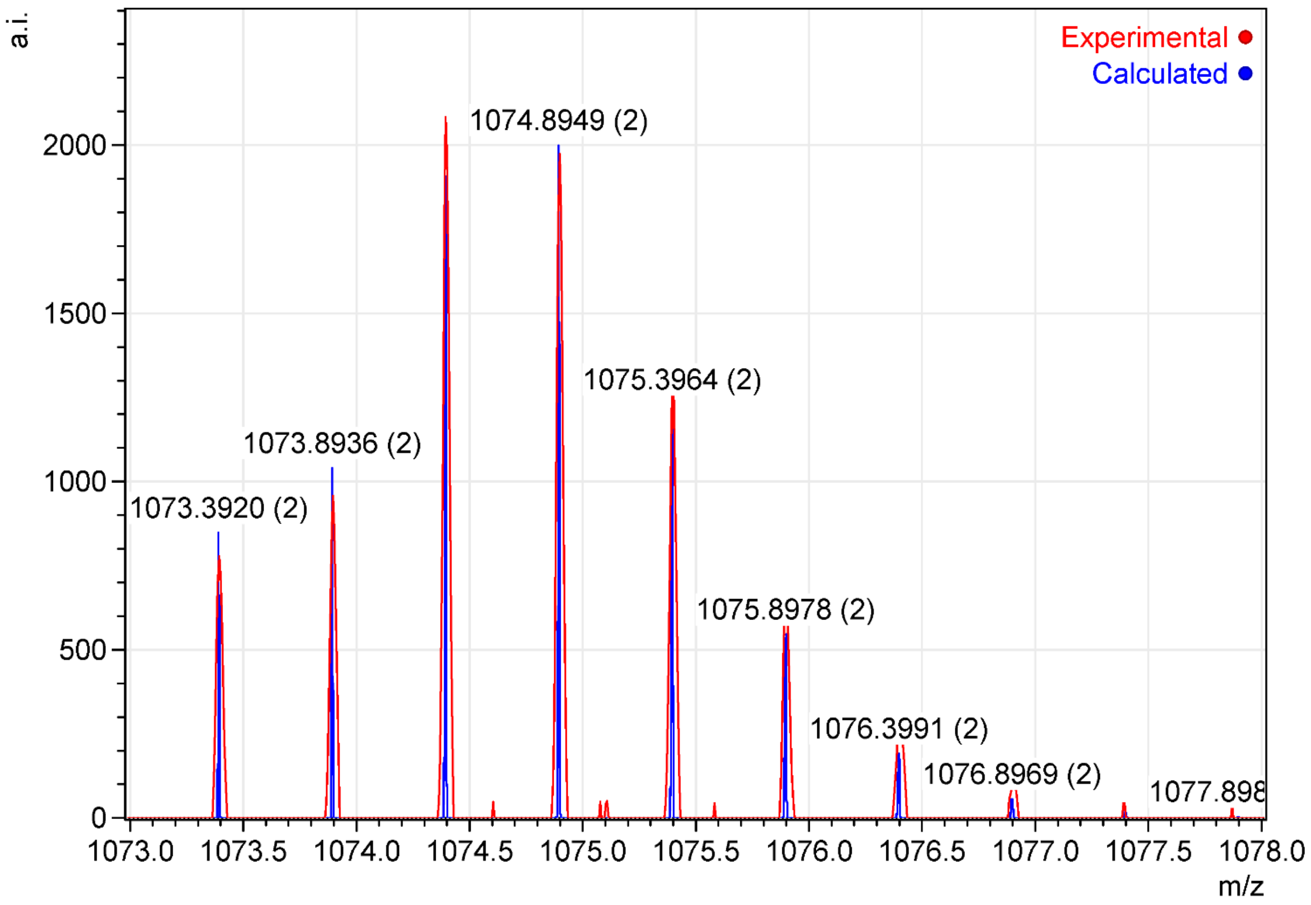

Figure S36. ESI^{+} mass-spectrum of **Ir1a** ($[M+H]^{2+}$ cation area), solvent – methanol.

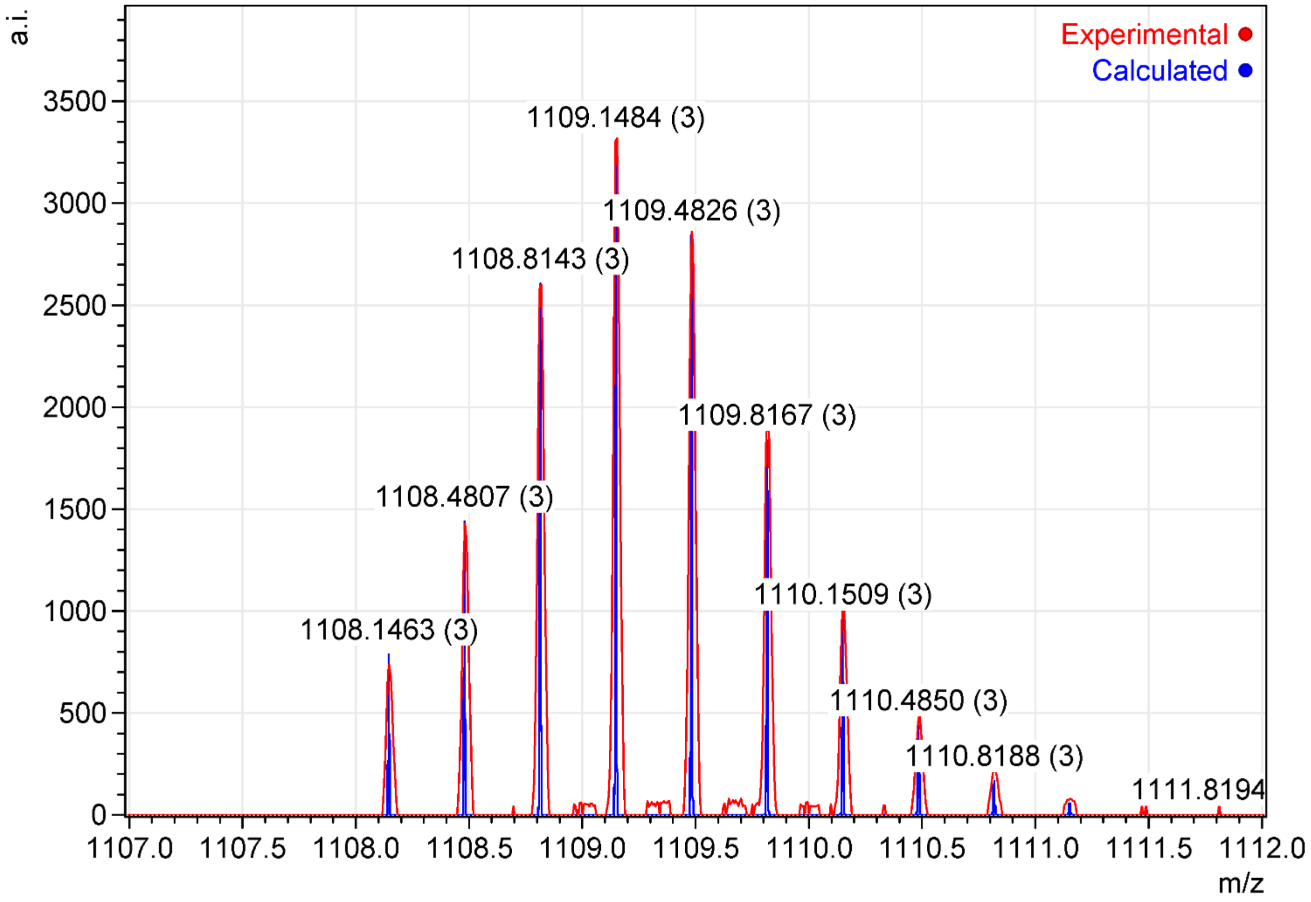

Figure S37. ESI$^+$ mass-spectrum of **Ir2a** ($[M+2H]^{3+}$ cation area), solvent – methanol.

Structural parameters

Table S1. Comparison of selected calculated bond lengths (*D*, Å) and angles (*A*, *deg*) for the ground state of complex **Ir0** and of a XRD data for closely analogous compound[1].

Parameters	Analogous Ir complex[1]	Parameters	Optimized **Ir0** singlet state
D(N1-Ir)	2.045	*D*(N5-Ir)	2.123
D(C13-Ir)	2.013	*D*(C44-Ir)	1.984
D(N2-Ir)	2.069	*D*(N7-Ir)	2.098
D(C26-Ir)	2.013	*D*(C52-Ir)	2.003
D(N3-Ir)	2.147	*D*(N3-Ir)	2.182
D(N4-Ir)	2.197	*D*(N4-Ir)	2.271
A(N1-Ir-N2)	176.64	*A*(N5-Ir-N7)	177.10
A(C13-Ir-N4)	171.96	*A*(C44-Ir-N2)	171.13
A(C26-Ir-N3)	175.25	*A*(C52-Ir-N3)	171.94

For atom numbering see Figure SS38.

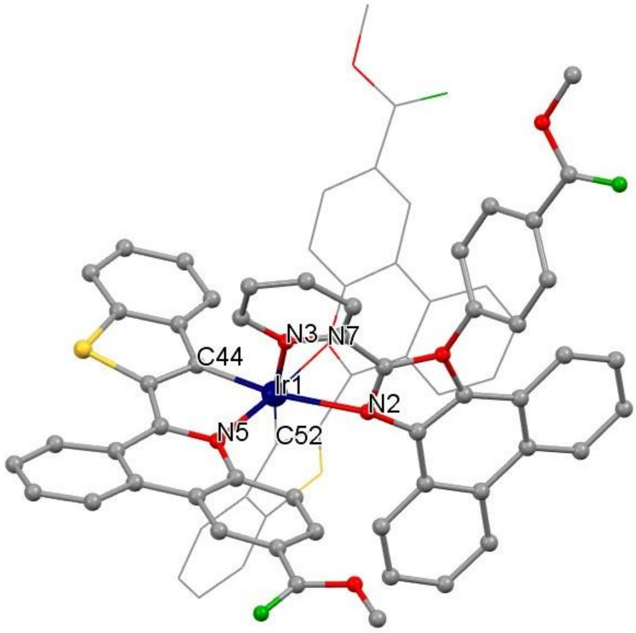

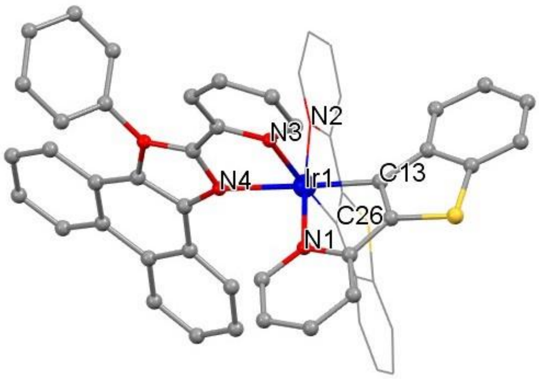

Figure S38. Optimized structures of model complex **Ir0** (top) and a closely analogous compound[1] (bottom). Atom colors: Ir – blue; N – red; S – yellow; O – green; C – gray. Hydrogen atoms are omitted for clarity.

Photophysical data for complexes Ir1, Ir2, Ir2a

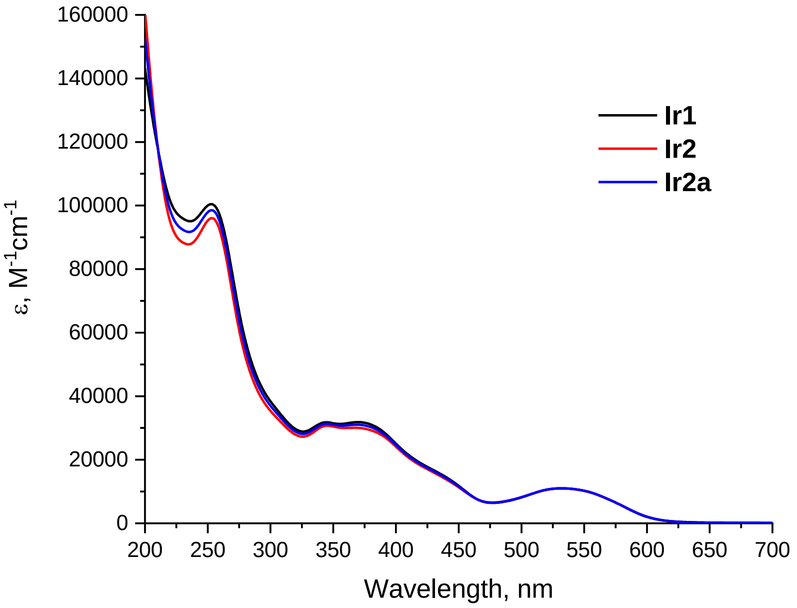

Figure S39. Absorption spectra of **Ir1**, **Ir2** and **Ir2a** in aqueous solution at 298K.

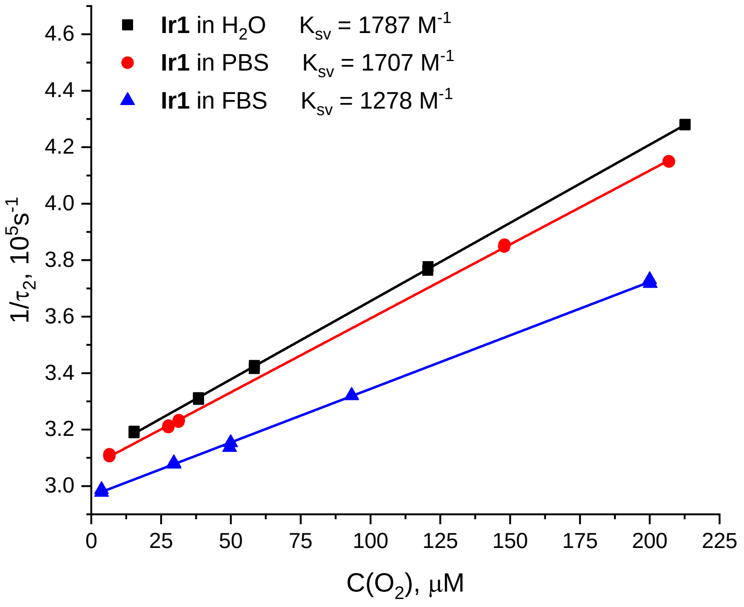

Figure S40. Stern-Volmer oxygen quenching plots of **Ir1** in aqueous solution (based on τ_2 measurements), 0.01M phosphate buffered saline (PBS) pH 7.4 and in fetal bovine serum (FBS), T = 37°C (K_{sv} - Stern-Volmer constant).

Table S2. Analysis of emission decay for the complex **Ir2** in H_2O, PBS and FBS solutions (T = 37°C).

	**C(O_2), μM**	**τ_1, ns**	**τ_2, ns**	**τ_1 contribution, %**
Solution in water	215.3	605	2158	17.0
	215.2	599	2161	16.5
	45	825	2924	14.1
	44.89	834	2926	14.7
	36.33	759	2989	13.4
	36.2	770	2990	13.8
	7.51	825	3177	12.9
	7.47	798	3180	12.3
	1.01	838	3235	13.0
	0.95	817	3236	12.5
Solution in PBS	203.3	617	2216	15.0
	203.1	627	2217	15.0
	147.7	640	2436	12.8
	147.5	649	2434	13.3
	32.36	781	3082	13.7
	32.3	798	3080	13.1
	10.57	867	3223	13.0
	10.5	843	3240	12.1
	5.54	855	3267	11.3
	5.47	829	3269	11.8
Solution in FBS	201.7	939	2540	21.7
	201.6	923	2541	21.9
	177.3	940	2607	18.8
	177.2	966	2608	18.9
	120	1014	2805	17.2
	119.7	979	2816	16.4
	33.1	1101	3163	11.4
	33.05	1007	3168	12.0
	7.94	977	3290	9.6
	7.89	982	3294	9.6

Table S3. Phosphorescence lifetime dependence on oxygen concentration for **Ir1**, **Ir2** and **Ir2a** H_2O, PBS and FBS solutions (T = 37°C).

	**Solution in water**		Solution in PBS		Solution in FBS	
**Complex**	**C(O_2), μM**	**τ_2, ns**	C(O_2), μM	τ_2, ns	C(O_2), μM	τ_2, ns
**Ir1**	212.7	2336	206.9	2410	200.2	2689
	212.6	2337	206.8	2409	200	2680
	120.6	2647	148	2594	93.28	3011
	120.5	2656	147.9	2598	93.21	3011
	58.43	2918	31.35	3092	49.94	3170
	58.4	2927	31.25	3097	49.59	3187
	38.46	3023	27.69	3111	29.69	3249
	38.41	3018	27.65	3116	29.58	3244
	15.37	3130	6.51	3219	3.7	3347
	15.34	3136	6.49	3212	3.68	3358
**Ir2**	215.3	2158	203.3	2216	201.7	2540
	215.2	2161	203.1	2217	201.6	2541
	45	2924	147.7	2436	177.3	2607
	44.89	2926	147.5	2434	177.2	2608
	36.33	2989	142.3	2466	168.5	2648
	36.2	2990	141.8	2465	168	2649
	7.51	3177	67.71	2833	120	2805
	7.47	3180	67.61	2832	119.7	2816
	1.01	3235	32.36	3082	63.28	3030
	0.95	3236	32.3	3080	63.05	3030
			10.57	3223	33.1	3163
			10.5	3240	33.05	3168
			5.54	3267	7.94	3290
			5.47	3269	7.89	3294
**Ir2a**	217.4	2131	203.2	2228	200.5	2568
	217.2	2128	202.8	2230	200.2	2582
	150.3	2384	157.1	2393	171.5	2650
	150	2376	156.9	2397	171.2	2661
	67.54	2816	143.6	2450	103.2	2892
	67.3	2801	143.5	2454	103	2901
	36.68	3006	43.59	2998	39.28	3146
	36.61	3001	43.43	3005	39.26	3160
	5.27	3222	34.55	3067	16.56	3290
	5.02	3230	34.4	3071	16.49	3281
			6.12	3273	10.89	3327
			6.01	3277	10.84	3328
			0.63	3335	0.69	3352
			0.51	3327	0.67	3358

Table S4. Ir2 phosphorescence lifetime (τ_2) variations in aerated aqueous solution, as a function of probe concentration and pH, (T = 37°C).

**$C_{(Ir2)}$**	**τ_2, ns**	**pH**	**τ_2, ns**
10^{-7} M	2080	4.0	2169
10^{-6} M	2116	5.1	2083
10^{-5} M	2095	6.0	2154
10^{-4} M	2133	7.0	2117
10^{-3} M	2076	8.1	2222

Table S5. Ir2 phosphorescence lifetime (τ_2) variations in aerated FBS solution as a function of serum concentration and dynamic viscosity (**Ir2** concentration = 10^{-5} M, T = 37°C).

$C_{(FBS)}$, %	τ_2, ns	Dynamic viscosity, mPa·s
0* (PBS)	2158	0,698
10	2327	0,724
30	2378	0,776
50	2434	0,838
70	2490	0,902
90	2522	0,977
100*	2540	1,018

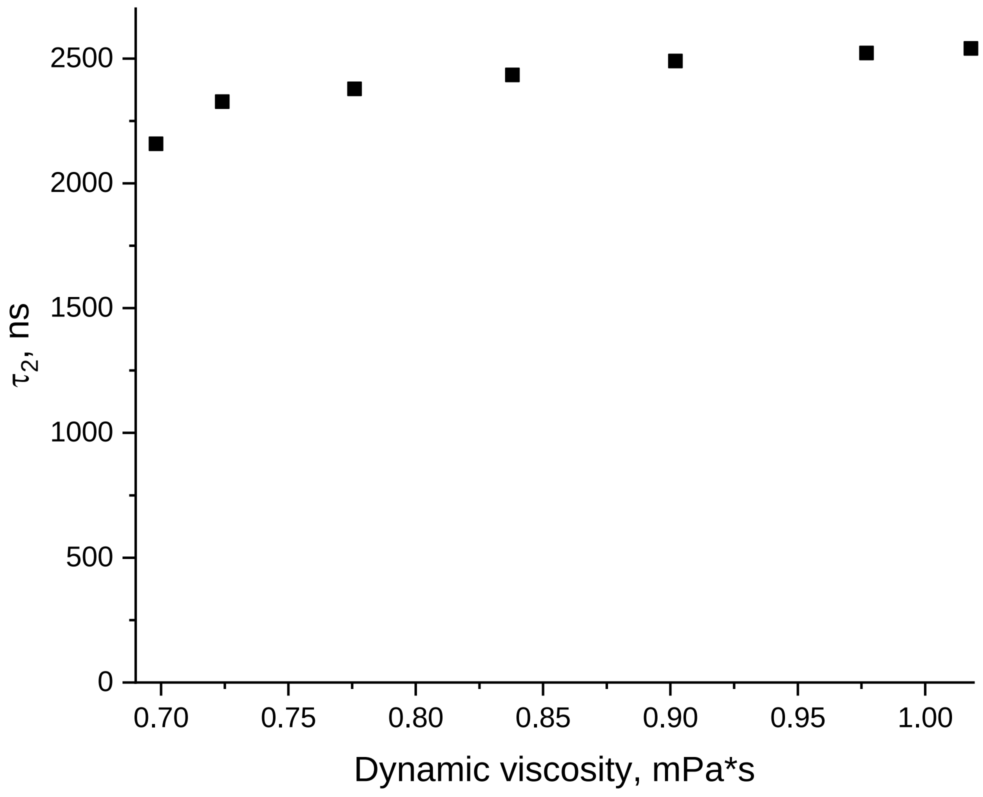

Figure S41. Ir2 phosphorescence lifetime (τ_2) dependence on dynamic viscosity values of water-FBS solutions (0, 10, 30, 50, 70, 90 and 99%, T = 37°C, λ_{exc} 351 nm).

Table S6. Comparison of the phosphorescence lifetime (τ_2) data for **Ir2** (10^{-5} M) obtained in aerated solutions in cuvette and using PLIM measurements with microscope.

	τ_2, ns PLIM	τ_2, ns cuvette
H_2O	2100	2160
PBS	2150	2220
FBS	2532	2540

Cell experiments

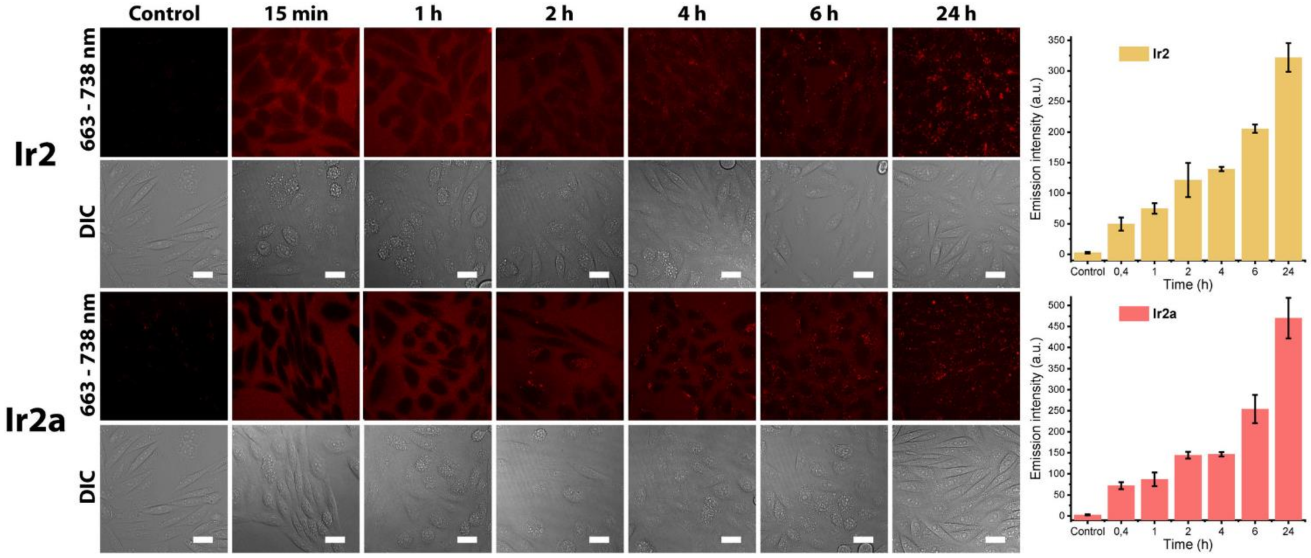

Figure S42. Dynamics of **Ir2** and **Ir2a** internalization into CHO-K1. On the left: Luminescence intensity and differential interference contrast (DIC) microscopy images of cells incubated with 40 μM of the dye for different periods of time. Scale bar 20 μm. Graphs on the right: Quantification of phosphorescence intensity inside the cells. Mean SD (n = 10 cells).

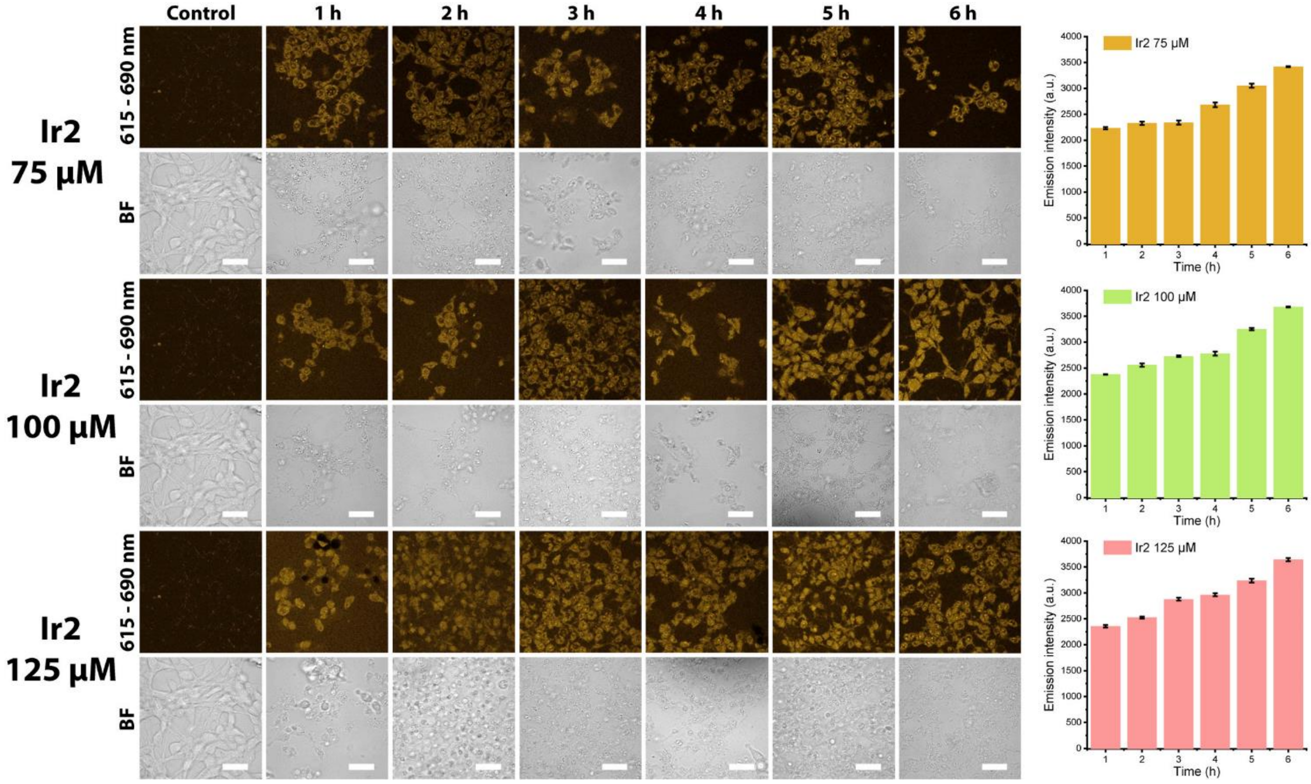

Figure S43. Dynamics of **Ir2** internalization into CT26 cells. On the left: Luminescence intensity and differential bright-field (BF) microscopy images of cells incubated with 75, 100, and 125 μM of the dye for different periods of time. Scale bar 40 μm. Graphs on the right: Quantification of phosphorescence intensity inside the cells. Mean SD (n = 10 cells).

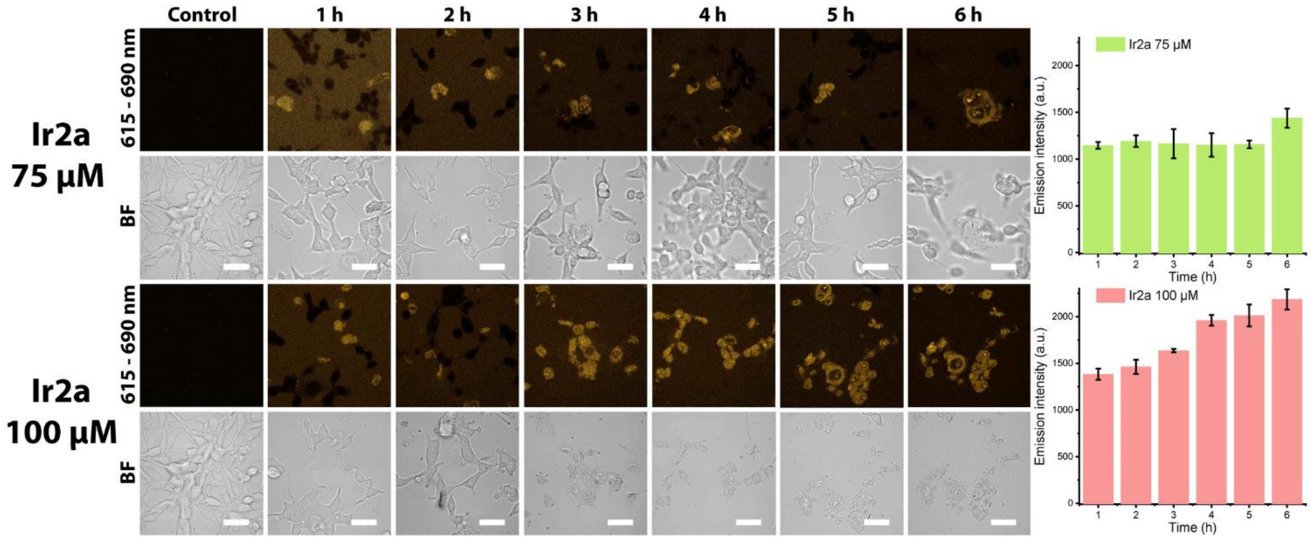

Figure S44. Dynamics of **Ir2a** internalization into CT26 cells. On the left: Luminescence intensity and differential bright-field (BF) microscopy images of cells incubated with 75 and 100 µM of the dye for different periods of time. Scale bar 40 µm. Graphs on the right: Quantification of phosphorescence intensity inside the cells. Mean SD (n = 10 cells).

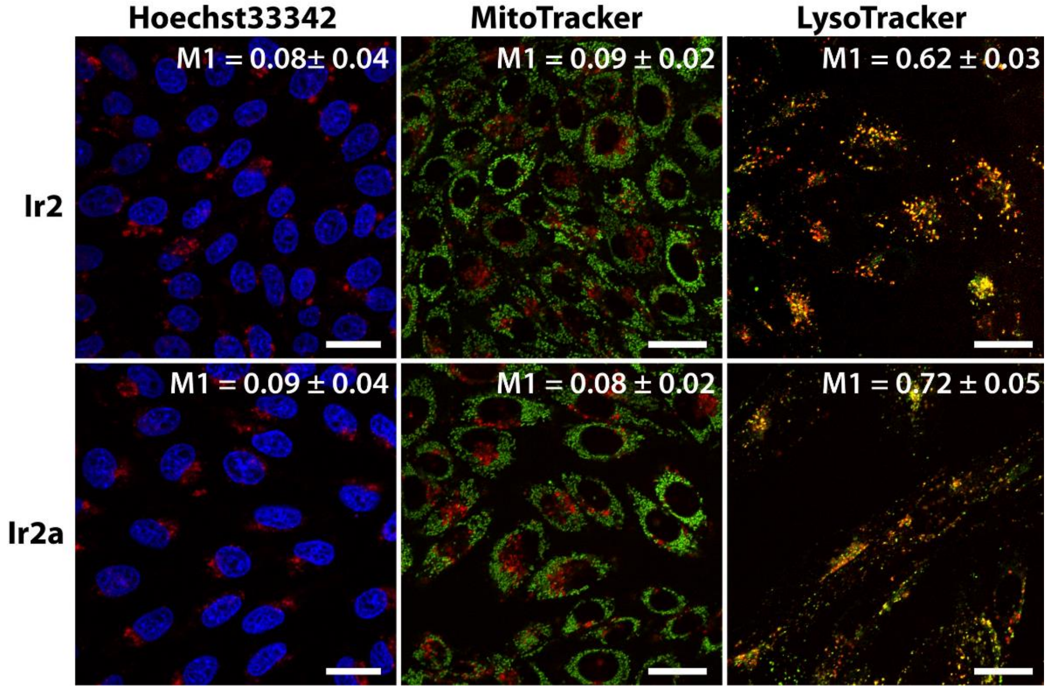

Figure S45. Subcellular distribution of complexes **Ir2** and **Ir2a** in CHO-K1 cells (red color of luminescence). Cells were co-stained with complexes and organelle-specific probes for nuclei - Hoechst33342 (blue color of luminescence), mitochondria - MitoTracker Green (green color of luminescence), and lysosomes - LysoTracker Green (green color of luminescence). Scale bars: 20 µm. Manders' overlap coefficients (M1) are shown in the pictures.

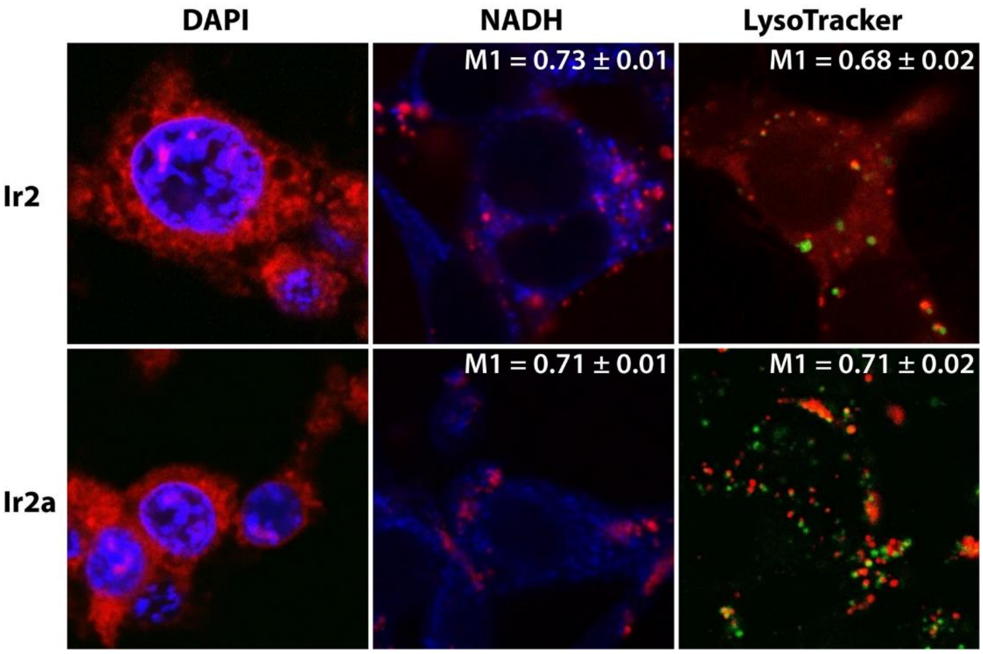

Figure S46. Subcellular distribution of complexes **Ir2** and **Ir2a** in CT26 cells (red color of luminescence). Cells were co-stained with complexes and organelle-specific probes for nuclei – DAPI (blue color of luminescence) and lysosomes - LysoTracker Orange (orange color of luminescence). Mitochondria were identified by autofluorescence of NADH (blue color of luminescence).

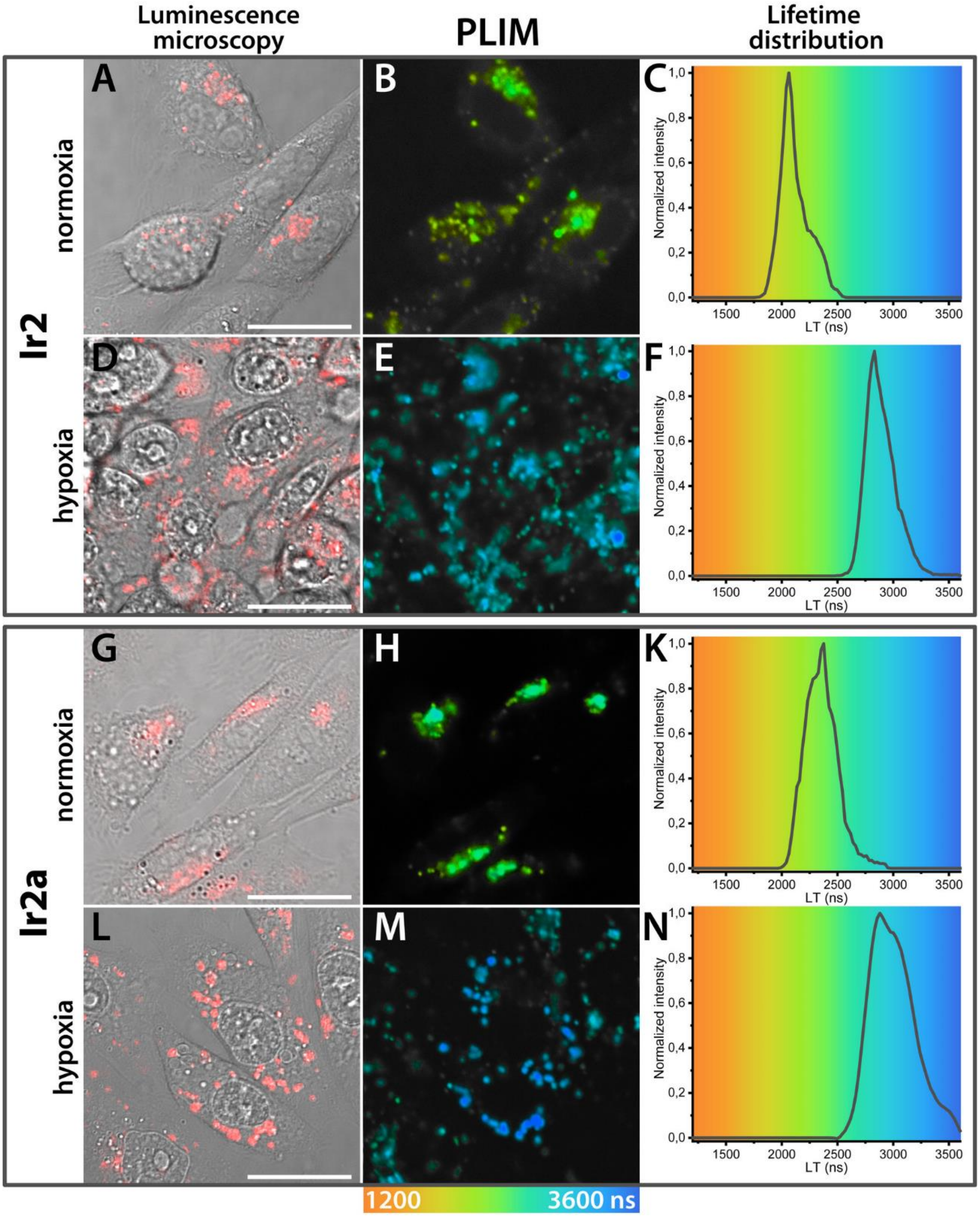

Figure S47. Phosphorescence lifetime distribution in CHO-K1 cells incubated with 40 μM of **Ir2** (A-F) and **Ir2a** (G-N) for 24h under normoxia (A-C, G-K) and hypoxia conditions (D-F, L-N). A, D, G, L – luminescent microscopy (excitation 405 nm, detection 663-738 nm) stacked with DIC-image. B, E, H, M – PLIM images (excitation 405 nm, detection 690-750 nm), colors corresponds to the lifetime in the range 1200-3600 nm. C, F, K, N – lifetime distribution for whole PLIM image.

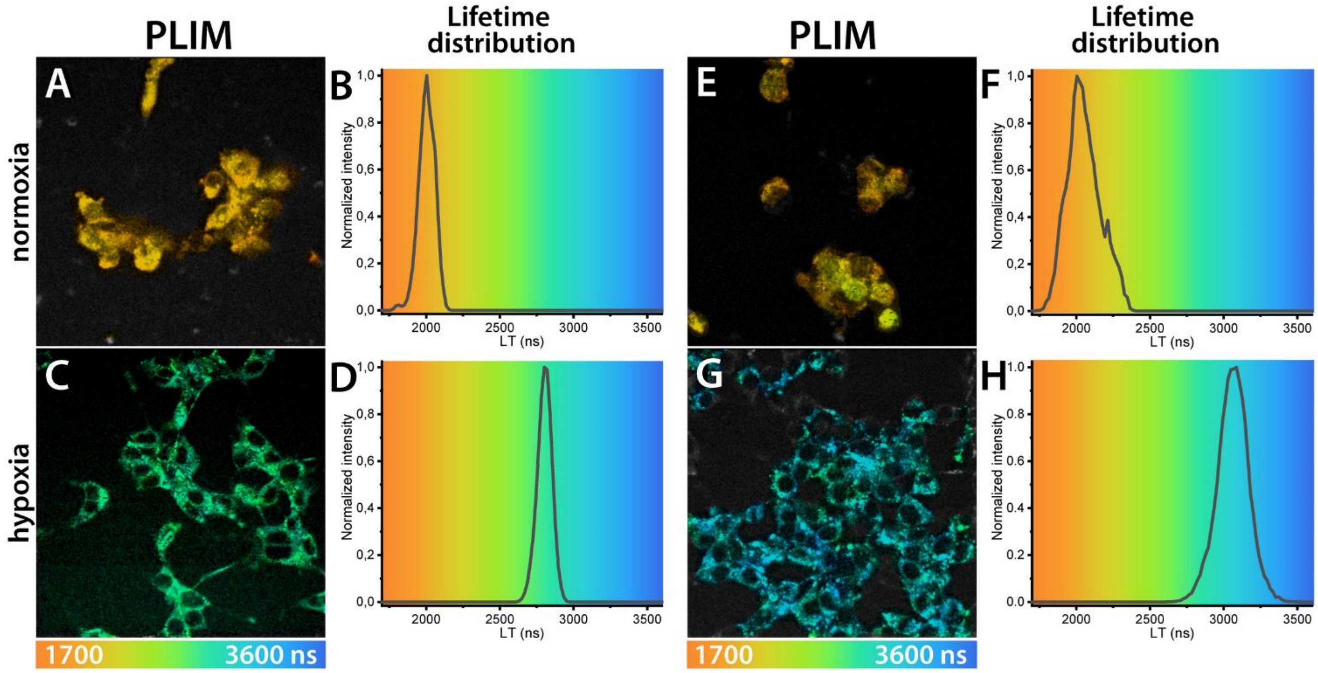

Figure S48. Phosphorescence lifetime distribution in CT26 cells incubated with 100 μM of **Ir2** (A-D) and **Ir2a** (E-H) for 24h under normoxia (A, B, E, F) and hypoxia conditions (C, D, G, H). B, C, E, G – PLIM images, colors corresponds to the lifetime in the range 1700-3600 nm. B, D, F, H – lifetime distribution for whole PLIM image.

References

1 A. I. Solomatina, K.M. Kuznetsov, V. V Gurzhiy, V. V Pavlovskiy, V. V Porsev, R.A., R.A. Evarestov and S. P. Tunik, *Dalt. Trans.*, 2020, **49**, 6751–6763.
